# Social capital II: determinants of economic connectedness

**DOI:** 10.1038/s41586-022-04997-3

**Published:** 2022-08-01

**Authors:** Raj Chetty, Matthew O. Jackson, Theresa Kuchler, Johannes Stroebel, Nathaniel Hendren, Robert B. Fluegge, Sara Gong, Federico Gonzalez, Armelle Grondin, Matthew Jacob, Drew Johnston, Martin Koenen, Eduardo Laguna-Muggenburg, Florian Mudekereza, Tom Rutter, Nicolaj Thor, Wilbur Townsend, Ruby Zhang, Mike Bailey, Pablo Barberá, Monica Bhole, Nils Wernerfelt

**Affiliations:** 1 Department of Economics, Harvard University, Cambridge, MA USA; 2 Department of Economics, Stanford University, Stanford, CA USA; 3 NYU Stern School of Business, New York, NY USA; 4 Opportunity Insights, Harvard University, Cambridge, MA USA; 5 Grammarly, San Francisco, CA USA; 6 Meta Platforms, Menlo Park, CA USA

**Keywords:** Economics, Sociology

## Abstract

Low levels of social interaction across class lines have generated widespread concern^1–4^ and are associated with worse outcomes, such as lower rates of upward income mobility^4–7^. Here we analyse the determinants of cross-class interaction using data from Facebook, building on the analysis in our companion paper^7^. We show that about half of the social disconnection across socioeconomic lines—measured as the difference in the share of high-socioeconomic status (SES) friends between people with low and high SES—is explained by differences in exposure to people with high SES in groups such as schools and religious organizations. The other half is explained by friending bias—the tendency for people with low SES to befriend people with high SES at lower rates even conditional on exposure. Friending bias is shaped by the structure of the groups in which people interact. For example, friending bias is higher in larger and more diverse groups and lower in religious organizations than in schools and workplaces. Distinguishing exposure from friending bias is helpful for identifying interventions to increase cross-SES friendships (economic connectedness). Using fluctuations in the share of students with high SES across high school cohorts, we show that increases in high-SES exposure lead low-SES people to form more friendships with high-SES people in schools that exhibit low levels of friending bias. Thus, socioeconomic integration can increase economic connectedness in communities in which friending bias is low. By contrast, when friending bias is high, increasing cross-SES interactions among existing members may be necessary to increase economic connectedness. To support such efforts, we release privacy-protected statistics on economic connectedness, exposure and friending bias for each ZIP (postal) code, high school and college in the United States at https://www.socialcapital.org.

## Abstract (web summary)

Social disconnection across socioeconomic lines is explained by both differences in exposure to people with high socioeconomic status and friending bias—the tendency for people to befriend peers with similar socioeconomic status even conditional on exposure.

## Main

Many researchers and policy-makers have raised concerns that societies around the world are fragmented and polarized, with little interaction across racial, political and class lines^1–4,8^. In addition to being of potential concern in its own right, a lack of interaction between different groups of people is associated with worse economic and social outcomes^4–6^. For example, in our companion paper^7^, we used data on social networks from Facebook to show that communities in which people with low SES interact less with people with high SES exhibit less upward income mobility across generations.

In this Article, we analyse the determinants of social interactions across class lines, examining why people with higher SES tend to have more high-SES friends. Building on previous research^9–11^, we distinguish two channels that can generate differences in an individual’s share of high-SES friends: (1) differences in high-SES exposure, the share of high-SES members in the groups to which people with different socioeconomic statuses belong (for example, their schools or religious organizations), and (2) differences in friending bias, the rate at which people befriend high-SES individuals conditional on the share of high-SES members in the group (that is, conditional on exposure).

Distinguishing between these two channels is critical for developing interventions to increase economic connectedness—if differences in exposure are central, then efforts to increase socioeconomic integration in neighbourhoods and schools may be the key to increasing connectedness; by contrast, if friending bias is central, one must instead focus on how to increase social interaction across class lines within existing neighbourhoods and schools.

We use data on the social networks of 70.3 million Facebook users between the ages of 25–44 years to construct and publicly release (https://www.socialcapital.org) privacy-protected measures of exposure and friending bias for each high school, college and ZIP code in the United States. As in previous research using Facebook data^7,12–16^, we use social network data as a proxy for real-world friendships rather than online interactions per se; as a result, our analysis does not shed light on the effects of online social networks themselves on exposure and friending bias.

We show that exposure and friending bias each account for about half of the social disconnection between people with low versus high SES. Like exposure, friending bias is shaped by institutional structure, such as the size of groups and the settings in which people interact. We demonstrate how the data we release publicly can be used to inform interventions to increase cross-class interaction by identifying where efforts to increase socioeconomic diversity versus reduce friending bias would be most valuable.

## Determinants of economic connectedness

Following our companion paper^7^, we measure the degree of cross-class interaction—which we term economic connectedness, or EC—as the share of friends with above-median SES (‘high SES’) among people with below-median SES (‘low SES’) divided by 50%, to quantify the average degree of under-representation of high-SES friends among people with low SES. A value of 0 for EC implies that a network has no connections between people with low and high SES, whereas a value of 1 implies that people with low SES have an equal number of low- and high-SES friends. On average, EC is 0.776 for people with low SES in the United States^7^. That is, high-SES friends are under-represented by 22.4% (1 − 0.776 = 0.224) among individuals with low SES relative to the high-SES share in the population. Our goal is to determine the factors that generate this 22.4% under-representation of high-SES friends.

Within any group in which friendships are made—such as a specific high school or neighbourhood—the rate at which people with low SES become friends with people with high SES depends on two factors: (1) high-SES exposure^17,18^, the share of high-SES members in the group; and (2) friending bias^11^, the rate at which people befriend high-SES individuals conditional on the share of high-SES members in the group (that is, conditional on exposure). For example, in the context of schools, students with low SES may have fewer friends with high SES because they attend schools with few high-SES students (that is, schools with low exposure) or because they are less likely to befriend high-SES students even within their schools (that is, their schools have high friending bias).

We measure exposure in a group as the share of individuals in the group with above-median SES multiplied by two, such that exposure is equal to 1 for a group in which 50% of individuals have above-median SES (Methods: ‘Decomposing EC’). Exposure is below 1 for groups that have a below-average share of individuals with high SES and above 1 for groups that have an above-average share of individuals with high SES.

We define a person’s friending bias in each group as one minus the share of friends they make in that group who have high SES divided by the share of people in the group who have high SES. If friendships were formed at random—and if people with high and low SES made the same number of friends—then individuals’ shares of friends with high SES in a given group would equal the share of individuals with high SES who belong to that group and friending bias would be equal to 0. Friending bias greater than 0 implies a lower probability of making high-SES friends than if friendships were formed at random within a given group.

In practice, high-SES individuals make more friends than low-SES individuals do on average^7^. Maintaining this difference in the number of total friends, in a setting with no homophily by SES (that is, where low- and high-SES individuals have the same probability of befriending a given person of high SES), friending bias would be negative; that is, the share of high-SES friends for the average low-SES individual would be greater than the share of high-SES people in the population. Quantitatively, given the empirically observed difference in the number of friends by SES, we would expect friending bias to be−11% in the absence of homophily (Methods: ‘Decomposing EC’).

Note that the distinction between exposure and friending bias depends on the level at which one measures exposure, and is therefore a policy-dependent rather than conceptual distinction. For example, friending bias in schools may arise from differences in high-SES exposure within schools due to tracking of students into different classrooms. Nevertheless, measuring exposure and friending bias at the school level has policy relevance because many interventions to increase socioeconomic integration, such as busing or changes in school attendance boundaries, focus on integration across rather than within schools (Supplementary Information C.1). Relatedly, the term friending bias should be interpreted in a statistical sense—denoting biased sampling from the pool of available peers—rather than as a normative statement about individuals’ preferences, as bias may be the result of institutional factors such as within-school tracking.

In addition to levels of exposure and friending bias within groups, economic connectedness also depends on where people make friends. For example, people with low SES are less likely to attend college and therefore make fewer friends in college than in other settings. As colleges tend to have many people with high SES and high levels of EC, low college attendance rates therefore reduce the EC of people with low SES (holding fixed exposure and friending bias at all colleges).

We measure exposure, friending bias and the share of friends that individuals make in six settings, comprising the most common places in which people make friends^19,20^: high schools, colleges, religious groups, recreational groups, workplaces and neighbourhoods. We estimate these measures separately by group (for example, separately for each high school in the United States) in each of these six settings using privacy-protected data from Facebook (Methods: ‘Sample construction’ and ‘Privacy and ethics’). As in our companion paper^7^, we focus on Facebook users aged between 25 and 44 years who reside in the United States, were active on the Facebook platform at least once in the previous 30 days, have at least 100 US-based Facebook friends and have a non-missing ZIP code. Here, we further restrict attention to individuals for whom we can allocate at least one friendship to the group in which it was formed, using the approach described in the ‘Variable definitions’ section of the Methods. The resulting sample consists of 70.3 million Facebook users, corresponding to 82% of the US population aged 25–44 years. On the basis of comparisons to nationally representative surveys and other supplementary analyses, we find that our Facebook analysis sample is reasonably representative of the national population^7^ (Methods: ‘Benchmarking’).

We use the Facebook data to obtain information on friendships, locations (ZIP code and county), own and parental socioeconomic status, and group memberships; we describe these variables in detail in the ‘Variable definitions’ section of the Methods. To capture the varied definitions of socioeconomic status used in previous work^21^, we compute socioeconomic status by combining several measures of SES, such as average incomes in an individual’s neighbourhood and levels of educational attainment. We combine these measures of SES into a single index using a machine learning algorithm described in the ‘Variable definitions’ section of the Methods and discussed further and validated against external measures in our companion paper^7^. We allocate each individual’s friendships to the groups in which they were formed using information from Facebook profiles and group memberships (Methods: ‘Variable definitions’).

### Exposure and friending bias by setting

We first analyse how rates of friendship formation, EC, exposure and friending bias vary across settings.

**Fig. 1:**
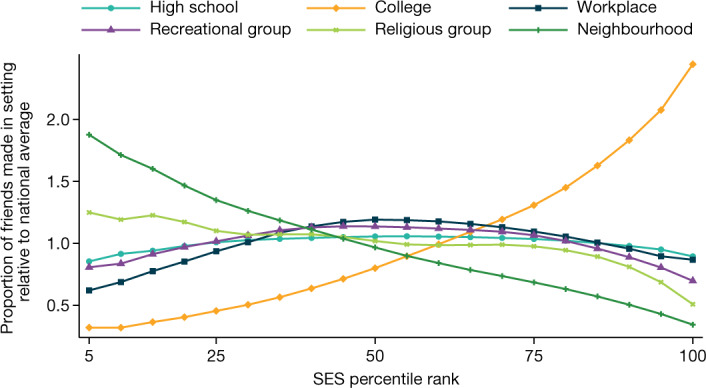
Friending rates by setting and SES. Friending rates across settings by the SES percentile rank of individuals in our primary analysis sample. The primary analysis sample consists of individuals between the ages of 25 and 44 years as of 28 May 2022 who reside in the United States, have been active on the Facebook platform at least once in the previous 30 days, have at least 100 US-based Facebook friends, have a non-missing residential ZIP code and for whom we are able to allocate at least one friend to a setting using the algorithm described in the ‘Variable definitions’ section of Methods. The vertical axis shows the relative share of friends made in each of the six settings that we analyse (for example, high schools), defined as the average fraction of friends made in that setting by people in a given SES ventile (5 percentile rank bin) divided by the fraction of friends made in that setting in the whole sample. Numbers above 1 imply that people at a given SES rank make more friends in a given setting than the average person; numbers below 1 imply the opposite. Extended Data Table 4 lists the underlying shares of friendships made in each setting for people with below-median SES versus above-median SES.

Figure 1 shows how the share of friends that an individual makes in each setting varies with their SES rank. For each SES ventile, it plots the average proportion of friends made in each setting, divided by the overall proportion of friends made in that setting across all SES ventiles. Individuals with the lowest SES make about four times greater a share of their friends in their neighbourhoods (residential ZIP codes) compared with individuals with the highest SES. By contrast, high-SES individuals make a far greater share of their friends in college than low-SES individuals do, primarily because individuals with high SES are much more likely to attend college. Neighbourhoods therefore play a larger role in defining the social communities of low-SES individuals, perhaps explaining why where one lives matters more for the economic and health outcomes of lower-income individuals than higher-income individuals^22,23^.

**Fig. 2:**
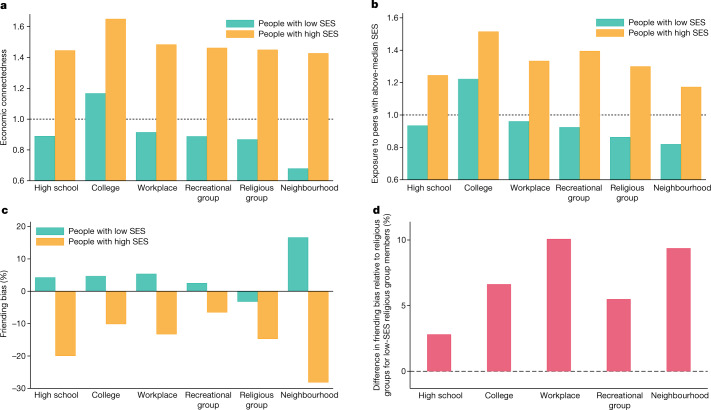
EC, exposure and friending bias by setting and SES. **a**–**d**, Variation in EC, exposure and friending bias across six settings where friendships are formed by individuals’ SES. All of the plots are based on the primary analysis sample defined in the legend of Fig. 1. **a**, Economic connectedness (EC) by setting for individuals with below-median SES (left, green bars) and above-median SES (right, orange bars). For both low- and high-SES individuals, EC is defined as twice the fraction of above-median-SES friends made within each setting. **b**, Mean rate of exposure to high-SES individuals in an individual’s group (for example, their high school) by setting for individuals with below-median SES (left, green bars) and above-median SES (right, orange bars). High-SES exposure is defined as two times the fraction of above-median-SES members of the individual’s group. **c**, Mean friending bias by setting for individuals with below-median SES (left, green bars) and above-median SES (right, orange bars). Friending bias is defined as one minus the ratio of the share of above-median-SES friends to the share of above-median-SES peers in the individual’s group. EC, high-SES exposure and friending bias are all calculated at the individual level and then aggregated to the setting × SES level (Supplementary Information B.5). **d**, Restricting the sample to low-SES members of religious groups, plots these individuals’ friending bias in each of the other settings minus their friending bias in religious groups. Extended Data Table 4 lists the values of average EC, bias and exposure shown in this figure.

Figure 2a shows how EC varies across the six settings for people with below- versus above-median SES. For each SES category, we define the setting-specific EC as two times the average share of friends made in that setting who have high SES. EC for people with low SES is highest in colleges and lowest in their residential neighbourhoods. However, even in colleges, low-SES people are much less likely to befriend high-SES people  than high-SES people are. To understand why, we next examine rates of high-SES exposure and friending bias in each setting.

Figure 2b shows average exposure to high-SES peers for low- and high-SES individuals across the six settings, among those who are assigned to a group in that setting. The exposure of individuals with low SES to high-SES peers is below 1 (that is, fewer than 50% of their peers have above-median SES) on average in all settings except in colleges, in which exposure is above 1 because most people who attend college have high SES. By contrast, for individuals with high SES, exposure to high-SES peers is well above 1 in all settings. This disparity in exposure reflects segregation across groups; for example, high-SES people tend to attend different religious institutions and colleges compared with low-SES people, as is well known from previous studies on segregation.

Social network data enable us to go beyond measures of segregation and analyse differences in rates of interaction conditional on exposure. This ability to identify interaction (friendship) rather than merely exposure (geographical proximity) is a key distinction between the present study and recent work that measures experienced segregation using location data from mobile devices^24–28^. Figure 2c shows mean rates of friending bias—the extent to which rates of friendship with high-SES individuals deviate from rates of exposure to high-SES individuals—across settings. The green bars show that levels of friending bias for individuals with low SES are typically positive, but differ substantially across settings.

Friending bias is highest on average in neighbourhoods, in which the mean friending bias for individuals with low SES is 0.17. That is, low-SES people befriend high-SES people in their ZIP codes at a 17% lower rate than would be the case if they were to befriend individuals with high SES in proportion to their presence in their ZIP codes. Friending bias may be high at the neighbourhood level partly because of residential segregation within ZIP codes that limits opportunities for contact and interaction between people with low and high SES.

Friending bias is lowest on average in religious groups, in which friending bias is −0.03, implying that low-SES people tend to form friendships with high-SES members of their religious groups at a rate that is slightly higher than the share of high-SES people in their religious groups. Friending bias is negative in religious groups because religious-group friendships do not exhibit substantial homophily by SES—a finding that is consistent with previous research using survey data^29^—and because high-SES people make more friends than low-SES people. Holding fixed exposure, people with low SES are about 20% more likely to befriend a given high-SES person in their religious groups than in their neighbourhoods—a large difference, comparable in magnitude to the 22.4% under-representation of high-SES friends on average among low-SES individuals^7^. Put differently, if friending bias in all settings was reduced by an amount equal to the difference in friending bias between neighbourhoods and religious groups, most of the disconnection between low-SES and high-SES individuals in the US would be eliminated.

Since religious groups are highly segregated by income, as shown in Fig. 2b, their low friending bias does not currently translate to a high level of EC (Fig. 2a). Efforts to integrate religious groups by SES may be particularly effective at increasing EC if friending bias remains low as they become more integrated. This assumption is not innocuous—as illustrated by the challenges faced in efforts to integrate college classrooms^30^—but it is bolstered by the fact that religious groups exhibit low levels of friending bias at all levels of exposure (Supplementary Fig. 1b).

Figure 2c (orange bars) shows that, across all settings, people with high SES are more likely to befriend their fellow high-SES group members (and correspondingly less likely to befriend low-SES group members) than would be expected based solely on the socioeconomic compositions of their groups. Again, there is sizable heterogeneity in friending bias across settings: high-SES people exhibit the most friending bias (in absolute terms) in neighbourhoods, and the least in recreational groups.

A natural question that arises from these differences in friending bias across settings is whether they are an attribute of the setting itself, or a reflection of the types of individuals who join that setting. For example, religious groups might be particularly good at fostering ties between low- and high-SES members, or it could be that individuals who participate in religious groups are more likely to form cross-SES ties across all settings. To distinguish between these explanations, Fig. 2d plots friending bias in each of the other five settings minus friending bias in religious groups for low-SES members of religious groups. Members of religious groups exhibit much more friending bias in all other settings than they do in religious groups, showing that the settings in which friendships form matter.

The fact that friending bias varies significantly across settings suggests that it is in substantial part determined by the nature of the institutions in which people interact—consistent with Blau’s theory of social structure^31^—rather than entirely determined by preferences. This result suggests that friending bias can potentially be changed through policy interventions (for example, by changing the structure of the groups in which people interact), much as the socioeconomic composition of groups can be influenced by policy (for example, through busing or affordable housing programs). Next, we analyse how important it is to reduce friending bias versus increase exposure to increase EC by examining the relative contributions of these factors in generating differences in connectedness.

### Decomposing connectedness by SES

We quantify how much of the difference in the share of high-SES friends between people with low versus high SES is due to differences in friending shares across settings, differences in exposure, and differences in friending bias by conducting counterfactual exercises that sequentially remove variation in each of these three dimensions (Methods: ‘Decomposing EC’). Conceptually, our goal is to determine how much of the difference in connectedness would remain if people with low SES made friends in different settings at the same rates as high-SES people (same friending shares); if they participated in groups with the same shares of high-SES members (same exposure); and if they made friends with high-SES peers at the same rates conditional on exposure as high-SES individuals do (same friending bias).

**Fig. 3:**
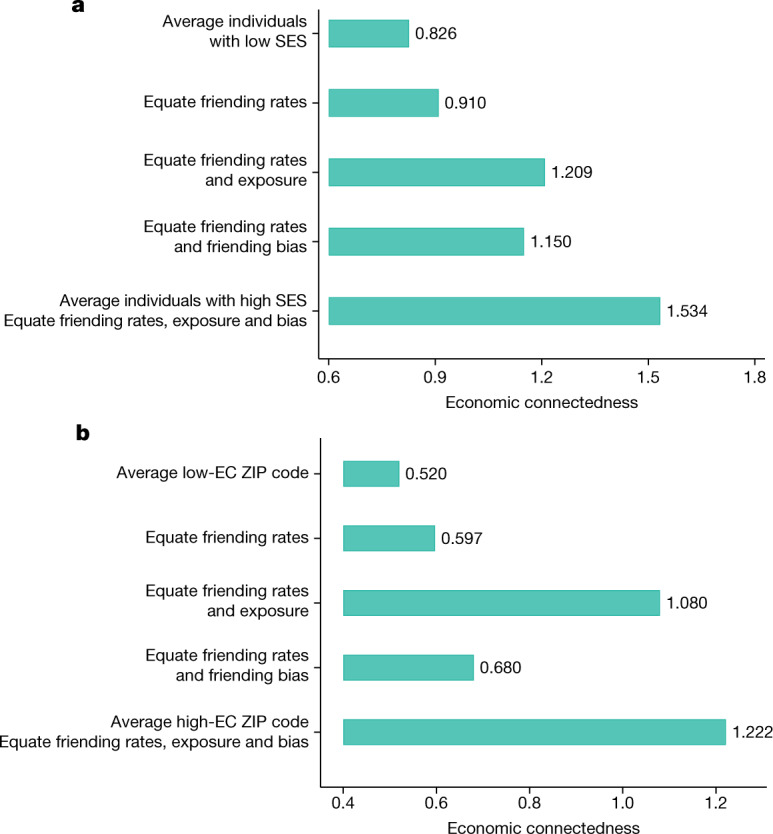
Determinants of differences in EC by SES and across ZIP codes. **a**, Share of the difference in EC between individuals with high versus low SES that is driven by differences in the settings in which they make friendships (friending rates), rates of exposure to individuals with high SES in those settings and friending bias conditional on exposure. The first and fifth bars show the observed EC for average low- and high-SES individuals, calculated as the EC for individuals who have setting-level friending rates, exposure rates and friending bias levels that match the means for low- and high-SES people in our sample, respectively (Methods: ‘Decomposing EC’). The middle three bars show the predicted EC for the average low-SES individual under various counterfactual scenarios. In the second bar, we consider a counterfactual scenario in which the friending rates across different settings for the average low-SES individual are equated to those of the average high-SES individual, while preserving exposure and friending bias at the mean observed levels for low-SES individuals within those settings. The third bar further equates the rate of high-SES exposure in each setting to match the observed mean values for high-SES individuals. The fourth bar equates rates of friending bias in each setting as well as friending rates across settings to match the observed mean values for high-SES individuals. The fifth bar equates rates of both exposure and friending bias within settings and friending rates across settings. **b**, A decomposition exercise analogous to **a** between ZIP codes with different levels of EC for below-median-SES residents instead of between individuals with below- versus above-median SES. The comparison of interest here is between ZIP codes in the bottom quintile of the EC distribution for below-median-SES residents (low-EC ZIP codes) and ZIP codes in the top quintile of EC for below-median-SES residents (high-EC ZIP codes). See Supplementary Information B.5 for further details on these counterfactual exercises.

Figure 3a presents the results of this exercise. The top bar shows that EC for the average low-SES individual is 0.83, whereas the bottom bar shows that EC for the average high-SES individual is 1.53—corresponding to a gap in EC by SES of 0.7 (Methods: ‘Decomposing EC’). Now consider equating the share of friends that the average low-SES person makes across the six settings to match that of the average high-SES person. Intuitively, this exercise examines what would happen to the EC of low-SES people if they were to make friends at the same relative rates across settings as high-SES people holding constant rates of exposure and friending bias across settings. For example, this counterfactual would increase the overall share of friends that low-SES people make in college to match that of high-SES people; however, it would not change the specific colleges that low-SES people attend to match those of high-SES people (as changes in groups within settings would generate a change in exposure).

The second bar in Fig. 3a shows that equating friending shares across settings by SES closes only 12% of the gap in EC between the average person with low versus high SES. Thus, differences in the settings in which people make friends explain little of why high-SES people have more high-SES friends. This is consistent with the fact that the variation in EC across settings for individuals with low SES is small compared with differences in EC by SES within each setting (Fig. 2a): even if low-SES individuals were to make all their friends in their highest-EC setting (colleges), their EC would still be substantially below that of high-SES individuals.

Next, we preserve these equated friend shares across settings and set the exposure rates in each setting for the average low-SES person to match the exposure rate for the average high-SES person in that setting. This counterfactual resembles a desegregation policy that adjusts the socioeconomic composition of groups but leaves friendship patterns within them unchanged. For example, in the context of colleges, this counterfactual can be interpreted as having students with low SES attend the same colleges as students with high SES, but retaining their current rate of befriending a given high-SES college peer. The third bar in Fig. 3a shows that equating exposure in addition to friending shares would increase the EC of the average low-SES individual to 1.21, closing 54% of the gap in EC between the average person with low versus high SES. Intuitively, this is because the gap in exposure by SES in Fig. 2b is approximately half as large as the gap in EC in Fig. 2a in most groups. Although a 54% reduction is substantial, it implies that even if neighbourhoods (ZIP codes), schools and colleges were perfectly integrated by SES, nearly half of the gap in EC between individuals with low and high SES would remain.

In the fifth bar, we further set friending bias in each setting for the average low-SES person equal to friending bias of the average high-SES person in that setting. Equating friending bias mechanically closes the remaining 46% gap in EC between the average person with low versus high SES.

### Decomposing connectedness across areas

**Fig. 4:**
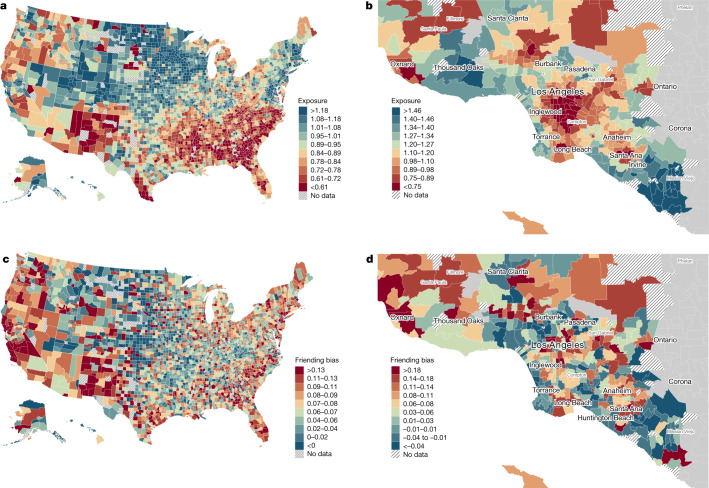
The geography of exposure and friending bias. **a**–**d**, Maps of mean high-SES exposure (**a**,**b**) and mean friending bias (**c**,**d**) for individuals with low SES. **a**,**c**, National county-level maps. **b**,**d**, ZIP-code-level maps of the Los Angeles metropolitan area. We aggregate individual-level statistics to compute ZIP-code-level and county-level means (Supplementary Information B.5). At the individual level, exposure is defined as the weighted average of two times the fraction of individuals with above-median SES in the groups in which an individual with below-median SES participates, weighting each group by the individual’s share of friends in that group. Friending bias is defined as one minus the weighted average of the ratio of the share of high-SES friends to the share of high-SES peers in the groups in which an individual with low SES participates, again weighting each group by the individual’s share of friends in that group. We use methods from the differential privacy literature to add noise to the statistics plotted here to protect privacy while maintaining a high level of statistical reliability; see www.socialcapital.org for further details on these procedures.

We use a similar approach to analyse why EC among people with low SES varies geographically^7^. We begin by collapsing our individual-level measures of exposure and bias to the county level, calculating mean high-SES exposure and friending bias among individuals with low SES for each county. Figure 4 maps these variables by county. Furthermore, we provide an illustrative example of local-area variation by presenting maps of these variables by ZIP code in the Los Angeles metropolitan area. As one might expect, exposure is generally higher in places with higher average incomes (Supplementary Information C.2), such as along each coast of the continental United States and near the coast in Los Angeles. Friending bias is lowest in the Midwest and Great Plains. Friending bias is lower on average in areas with more high-SES exposure, with a correlation of about −0.2 across counties, but there are many exceptions to this pattern. For example, the northeast generally has high exposure but also high friending bias (that is, people with low and high SES in the northeast are relatively well integrated in schools and neighbourhoods, but tend to befriend each other at lower rates).

We use these area-level statistics to decompose the sources of the ZIP-code-level variation in the EC of individuals with low SES (Methods: ‘Decomposing EC’). The top bar in Fig. 3b shows that the average EC for people with low SES living in ZIP codes that are in the bottom quintile of the national distribution of ZIP-code-level low-SES EC averages is 0.52. The bottom bar shows that the corresponding value for people with low SES living in the top quintile of ZIP codes (again in terms of average levels of EC among individuals with low SES) is 1.22. The bars in the middle decompose this top-to-bottom-quintile difference in EC by sequentially equating the share of friends made in different settings, rates of exposure to high-SES peers and rates of friending bias of the average low-SES person in bottom-EC-quintile ZIP codes to match the corresponding values for the average low-SES person in top-EC-quintile ZIP codes (Supplementary Information B.5). We find that 73% of the difference in EC between ZIP codes in the bottom and top quintiles of the EC distribution is explained by differences in exposure, while 16% is explained by differences in friending bias and 11% by differences in friending rates across settings.

The geographical variation in EC is driven primarily by differences in exposure because high SES exposure varies more at the geographical level, whereas friending bias varies more across settings (for example, between neighbourhoods and religious groups). The variation in exposure is 3.3 times greater across counties than across settings (Extended Data Table 1). By contrast, the variation in friending bias is 3.3 times greater across settings than across counties. Intuitively, in areas in which the share of people with high SES is high in one setting (for example, in neighbourhoods), it is generally high in other settings as well (for example, in schools). By contrast, friending bias tends to be relatively consistent by setting across geographies, with low-bias settings in one area (for example, religious groups) generally exhibiting low friending bias in other areas as well. In short, where one lives influences one’s exposure to individuals with high SES, but the groups in which one participates substantially shape the extent to which one interacts with those high-SES peers.

In summary, differences in high-SES exposure generate most of the variation in the EC of people with low SES across areas, but friending bias and exposure contribute about equally to explaining the difference in the share of high-SES friends between low- and high-SES people. The reason is that exposure varies more across areas than it does by individual socioeconomic status, whereas friending bias differs sharply by SES and is relatively stable (but large) across areas.

## Exposure, bias and upward mobility

Given that both exposure and friending bias contribute to differences in EC, we next examine whether the strong correlation between EC and upward income mobility documented in the companion paper^7^ is driven by one or both of these components. We define upward mobility as the average income rank in adulthood of children who grew up in families at the 25th percentile of the national income distribution in a given county or zip code, drawing on data from the Opportunity Atlas^23^.

In column 1 of Table 1, we regress log[upward mobility] on log[EC] across ZIP codes (Methods: ‘Exposure, bias and upward mobility’). We find an elasticity of upward mobility with respect to EC of 0.24: a 10% increase in EC is associated with a 2.4% increase in upward mobility. In column 2, we regress log[upward mobility] on log[exposure] and log[1 − friending bias]. We find strong associations between both exposure and friending bias and measures of upward mobility, with elasticities of 0.25 and 0.19, respectively. Next, we examine how these relationships vary within versus across counties. Columns 3 and 4 of Table 1 include county fixed effects in the specifications from columns 1 and 2. When comparing ZIP codes within counties, higher exposure and lower friending bias remain strongly associated with higher levels of economic mobility, with elasticities of just under 0.25. In columns 5 and 6, we conversely focus on across-county variation by replicating columns 1 and 2 at the county level. We find qualitatively similar effects, although the estimates of the effects of friending bias on economic mobility become less precise, largely because most of the variation in friending bias is within rather than across counties (Extended Data Table 1).

**Table 1:** Associations between friending bias, exposure and upward income mobility across areas

Dependent variable	log[upward income mobility]						log[causal upward income mobility]
	ZIP codes				Counties		
	(1)	(2)	(3)	(4)	(5)	(6)	(7)
log[EC]	0.236***		0.227***		0.272***		
	(0.01)		(0.01)		(0.02)		
log[high-SES exposure]		0.248***		0.224***		0.286***	0.116***
		(0.01)		(0.02)		(0.03)	(0.02)
log[1 − friending bias]		0.185***		0.236***		0.142*	0.339***
		(0.03)		(0.04)		(0.08)	(0.07)
County FEs	No	No	Yes	Yes	No	No	No
Observations	24,200	24,200	24,200	24,200	2,986	2,986	2,136
*R*^2^	0.42	0.43	0.71	0.71	0.38	0.39	0.03

Estimates from OLS regressions of log[upward income mobility] on log[EC] and other covariates. The coefficients can be interpreted as the elasticity of upward mobility with respect to the relevant covariate. In columns 1–6, upward income mobility is obtained from the observational measures in the Opportunity Atlas^23^, and is defined as the predicted household income rank in adulthood for children with parents at the 25th percentile of the national income distribution. Columns 1–4 present regressions at the ZIP-code level. In column 1, the only independent variable is log[EC], defined here as the product of mean high-SES exposure and 1 − mean friending bias in the ZIP code (Supplementary Information B.5). In column 2, the independent variables are the log of mean high-SES exposure and the log of 1 − mean friending bias (Supplementary Information B.5). We start from individual-level statistics to compute ZIP-code-level and county-level means of exposure and friending bias (Supplementary Information B.5). At the individual level, exposure is defined as the weighted average of two times the fraction of above-median-SES members of the groups in which an individual with below-median SES participates, weighting each group by the individual’s share of friends in that group. Friending bias is defined as one minus the weighted average of the ratio of the share of friends with high SES to the share of peers with high SES in the groups in which a low-SES individual participates, again weighting each group by the individual’s share of friends in that group. Columns 3 and 4 replicate columns 1 and 2 adding county fixed effects. Columns 5 and 6 replicate columns 1 and 2 at the county level instead of ZIP-code level. Column 7 replicates column 6 using counties’ causal effects on upward mobility, defined as the mean predicted household income rank in adulthood for children with parents at the 25th percentile of the income distribution overall in the United States plus 20 times the raw annual causal exposure effect of growing up in the county reported in ref. ^32^. Regressions in columns 1-6 are weighted by the number of individuals in the primary analysis sample with below-median SES in the county or ZIP code. The regression in column 7 is weighted by the inverse of the squared standard error of the estimated annual causal exposure effect of growing up in that county^32^. Standard errors (reported in parentheses) are clustered at the commuting-zone level for county-level regressions and at the county level for ZIP-code-level regressions. Asterisks indicate the level of significance; *10%, **5%, ***1%.

In column 7 of Table 1, we change the dependent variable in the regression to the log of each county’s causal effect on upward mobility as estimated by Chetty and Hendren based on analysing movers^32^ (see the companion paper^7^ for further details on the interpretation of these causal effect measures). Both exposure and friending bias remain strongly predictive of counties’ causal effects on upward mobility, implying that moving to a place with greater exposure or lower friending bias at an earlier age increases the earnings in adulthood of children who grow up in low-income families.

We conclude that the relationship between economic connectedness and upward mobility is not driven merely by the presence of high-SES peers (for example, through the availability of additional resources for schools financed by local property taxes). Instead, interaction with those peers is what predicts upward mobility most strongly (see Supplementary Information C.3 for further discussion). In the context of schools, this result implies that the average income of classmates predicts upward mobility for low-SES students insofar as it affects the extent of their social interactions with high-SES students. Combined with our finding that friending bias accounts for around half of the difference in the share of high-SES friends between people with low versus high SES, these results imply that increasing EC—the form of social capital most strongly associated with economic mobility—would require efforts to both increase integration (exposure) and reduce friending bias within groups. In the next section, we show how our data can inform which of these approaches is likely to be most effective in a given group.

## Exposure and friending bias by high school

Having shown how exposure and friending bias vary across settings and areas, we now analyse variation in these statistics across the groups that comprise a given setting (for example, each high school in the ‘high school’ category). We begin by examining variation across high schools and then turn to variation across colleges. We publicly release estimates of exposure and friending bias for each high school and college as well as by neighbourhood (ZIP code); for religious organizations, recreational groups and employers, sample sizes are too small to obtain reliable estimates at the group-specific level.

For high schools, we report estimates based both on students’ own (post-high-school) SES in adulthood—the same SES measure that was analysed above—as well as estimates based on their parents’ SES (Methods: ‘High school estimates’). These measures have different applications. Measures of EC based on parental SES are relevant for policy discussions at the school level, which often focus on the degree of connection between children from different parental backgrounds. Measures based on own SES are useful for understanding the environments in which friendships between low-SES and high-SES adults are formed, that is, the extent to which a school might contribute to levels of EC in the next generation. Although the two measures capture different concepts, they yield fairly similar rankings of schools in terms of exposure and friending bias: the correlation between the two measures is 0.84 for exposure and 0.59 for friending bias across schools (Supplementary Table 1). We therefore focus on the parental SES measure here and present analogous results using own SES in Supplementary Fig. 2.

**Fig. 5:**
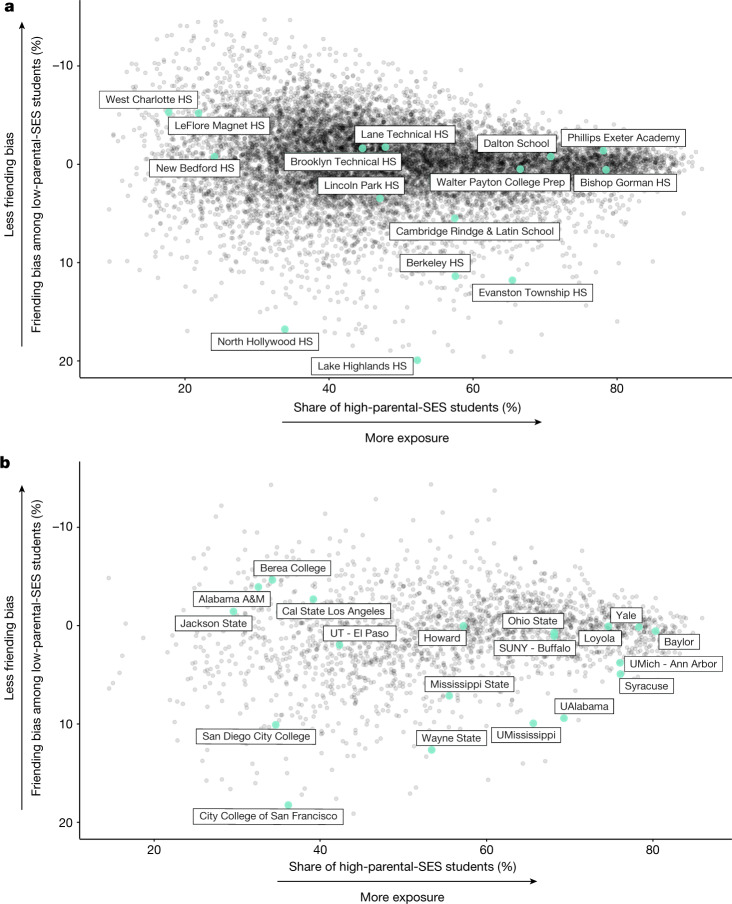
Friending bias and exposure by high school and college. **a**,**b**, Mean friending bias among students with low parental SES versus the share of students with high parental SES by high school (**a**) and college (**b**). Friending bias is defined as one minus the mean ratio of the share of high-school friends with high parental SES to the share of high-school peers with high parental SES, averaging over students with low parental SES (Supplementary Information B.5).The vertical axis is reversed, so that schools and colleges in the upper half of each panel have lower friending bias. The sample consists of individuals in the 1990–2000 birth cohorts (approximately spanning the high school and college graduating classes of 2008–2018 and 2012–2022, respectively) who could be linked to a specific school or college and to parents with an SES prediction. We report statistics only for high schools and colleges that have at least 100 low-SES and 100 high-SES Facebook users summing across these cohorts. We use methods from the differential privacy literature to add noise to the statistics plotted here to protect privacy while maintaining a high level of statistical reliability; see https://www.socialcapital.org for further details on these procedures. In this figure, SES refers to the SES of the individuals’ parents; Supplementary Fig. 2 replicates these figures using individuals' own (post-high school and post-college) SES ranks in adulthood.

Figure 5a plots friending bias (with an inverted vertical scale, so that moving up corresponds to less bias) against the share of students with high parental SES (that is, half of high-SES exposure) by high school. Both exposure to students with high parental SES (socioeconomic composition) and friending bias vary substantially across schools. The reliability of the exposure estimates, estimated using a split-sample approach (Methods: ‘High school estimates’), is 0.99 at the school level; that is, 99% of the variance in exposure reflects true differences in the share of students with high parental SES rather than sampling error. The reliability of the friending bias estimates is 0.58. This implies that a school that we estimate to have a 10% higher friending bias estimate will, on average, exhibit 5.8% higher bias in future cohorts. Estimates of exposure and friending bias based on own SES have higher reliabilities (0.99 for exposure and 0.88 for friending bias) because they use the full sample rather than just the subset of individuals that we can link to their parents.

Friending bias differs considerably even among nearby schools with similar socioeconomic compositions. For example, Walter Payton College Preparatory High School (‘Payton’) and Evanston Township High School (‘ETHS’) are two large high schools in the Chicago metro area that have similar fractions of students from families with above-median SES. However, ETHS has much higher friending bias than Payton: low-SES students at ETHS are much less likely to befriend their high-SES peers than low-SES students at Payton are, consistent with previous ethnographic evidence documenting high levels of friending bias at ETHS (Supplementary Information C.4). One potential explanation for this difference is the greater similarity of students on other dimensions at Payton relative to ETHS. Payton is a public magnet school that requires that all students complete an entrance exam. By contrast, ETHS is open to all students residing in the local catchment area, resulting in a more heterogeneous student body in terms of academic preparation—and concomitant segregation of classes—that may lead to higher friending bias^33^.

### Predictors of friending bias

**Extended Data Fig. 1:**
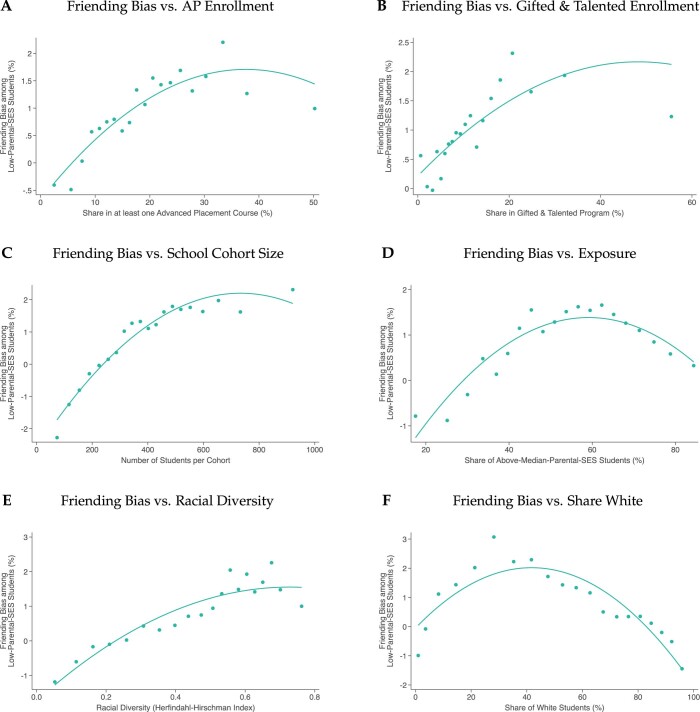
Predictors of Friending Bias in High Schools Using Parental SES. This figure shows school-level binned scatter plots of the average degree of friending bias (based on parental SES) among low-SES individuals versus various school-level characteristics: the share of students enroled in at least one Advanced Placement course (Panel A); the share of students enroled in a Gifted and Talented program (Panel B); total number of students per cohort (Panel C); the share of above-median-parental-SES students (Panel D); an index of racial diversity defined as $(1-{\Sigma }_{i}{s}_{i}^{2})$, where *s*_*i*_ is the fraction of race/ethnicity *i* (Black, white, Asian, Hispanic, Native American) in the school (Panel E); and the share of white students (Panel F). Friending bias is defined as one minus the mean ratio of the share of high-SES high school friends to the share of high-SES high school peers, averaging over low-SES students in the 1990-2000 birth cohorts (see Supplementary Information B.5). Shares of students enroled in at least one AP course and in a Gifted and Talented program are obtained from the 2015–2016 Civil Rights Data Collection (CRDC). School size and racial composition data are from the 2017–2018 National Center Education Statistics (NCES) data (see Supplementary Information A.2). The set of schools used in these plots is the same as in Fig.5, conditional on being present in the CRDC data. To construct the binned scatter plots, we divide the variable on the horizontal axis into ventiles (5 percentile point bins) and plot the mean of the vertical-axis variable against the mean of the horizontal-axis variable in each ventile. All binned scatter plots are weighted by the number of students in each high school as reported in the NCES data. As a visual guide to approximate the non-parametric relationships, the solid lines in each figure show lines of best fit from quadratic regressions estimated using OLS.

Building on this comparison, we examine the factors that predict friending bias across high schools more systematically by correlating bias across schools with various observable characteristics. Consistent with the ETHS–Payton comparison, we find that friending bias is higher on average in schools with more academic tracking as measured by enrolment rates in advanced placement and gifted and talented classes (Extended Data Fig. 1a,b). Friending bias is generally lower in smaller schools (Extended Data Fig. 1c), consistent with previous work documenting less homophily in smaller groups^31,34,35^.

In Extended Data Fig. 1d, we examine the relationship between friending bias and a school’s share of students with high parental SES. This relationship is non-monotonic, with friending bias highest in schools with an approximately equal representation of students from families with below- and above-median SES. This may be because there is less scope for low-SES or high-SES students to develop homogeneous cliques when there are relatively few members of their own group. Friending bias is also higher in more racially diverse schools, as measured by a Herfindahl–Hirschman index (Extended Data Fig. 1e) or the share of white students in the school (Extended Data Fig. 1f). One potential explanation for the link between racial diversity and friending bias by SES is that, when low- and high-SES students have different racial and ethnic backgrounds, they are less likely to be friends.

**Extended Data Fig. 2:**
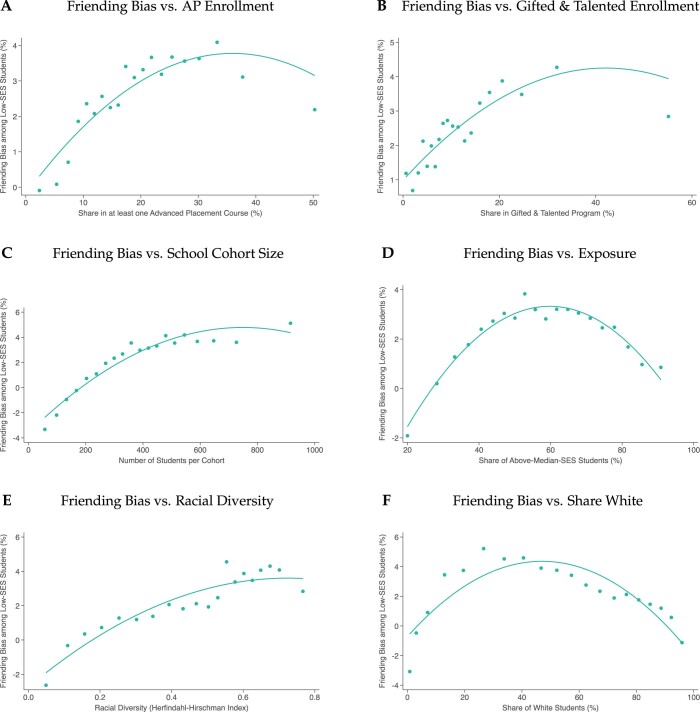
Predictors of Friending Bias in High Schools Using Own SES. This figure replicates Extended Data Fig. 1 using own SES rank in adulthood instead of parental SES rank when measuring friending bias and when defining the share of above-median SES students in Panel D. See notes to Extended Data Fig. 1 for further details.

**Extended Data Fig. 3:**
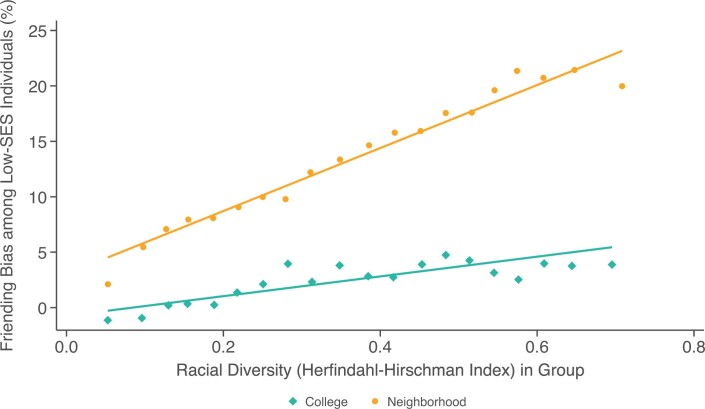
Friending Bias versus Racial Diversity. This figure presents binned scatter plots of friending bias against racial diversity within colleges (green diamonds) and neighbourhoods (ZIP codes, orange circles). See notes to Extended Data Fig. 1 for details on construction of binned scatter plots. We define racial diversity as $(1-{\Sigma }_{i}{s}_{i}^{2})$, where *s*_*i*_ is the fraction of race/ethnicity *i* (Black, white, Asian, Hispanic, Native American). Friending bias is defined as the average among below-median-SES individuals in the group (i.e., college or neighbourhood) of one minus the ratio of the share of above-median-SES friends in each individual’s group to the share of above-median-SES peers in that group. For comparability, both series measure SES using own SES rank in adulthood and use data from the 1986–1996 birth cohorts. Racial shares for each college and ZIP code are obtained from the 2013 Integrated Post-Secondary Education Data System (IPEDS) and the 2018 American Community Survey (ACS), respectively.

There are similar associations between these factors and friending bias between students who go on to have different socioeconomic statuses in adulthood themselves (Extended Data Fig. 2). In particular, in smaller and less racially diverse schools, there are more friendships between students who go on to have low and high SES in adulthood. We also find similar relationships between friending bias and group characteristics in other settings: higher levels of friending bias are associated with greater racial diversity across colleges and neighbourhoods (Extended Data Fig. 3) and larger group sizes across all six settings (Supplementary Fig. 1).

The explanatory factors considered in Extended Data Figs. 1–3 are not intended to be exhaustive, and much more remains to be learned about the determinants of friending bias. The main lesson we draw from these correlations is that, much like exposure, friending bias appears to be related to structural factors that can potentially be changed by policy interventions, such as reducing the size of groups and redesigning the nature of academic tracking within schools.

### Increasing connectedness

The variation in exposure and friending bias across schools documented in Fig. 5a implies that the most effective approach to increasing EC differs across schools. To increase EC in schools in the bottom half of Fig. 5a—such as Evanston Township High School, Berkeley High in Berkeley, CA, or Lake Highlands High in Lake Highlands, TX—decreasing friending bias (that is, increasing social interaction between students from different backgrounds) is likely to be valuable. For example, reducing friending bias at ETHS to zero would result in an increase of 0.15 (15 percentage points) in EC (measured in terms of parental SES). To benchmark this impact, note that the average parental EC among high school friends of individuals with low-SES parents across the schools in Fig. 5a is 0.92. This implies that, in the average US high school, students with low parental SES have 8% fewer high-parental-SES friends than one would expect in a scenario where students with high and low SES made the same total number of high school friends and exhibit no homophily. The current level of friending bias at ETHS therefore reduces the share of high-SES friends among low-SES students by almost twice the degree of under-representation of high-SES friends among students from low-SES families at the average US high school (15% versus 8%). Thus, at schools like ETHS, increasing cross-SES interaction within the student body may be a more effective way to increase EC than attempting to further diversify the student body. By contrast, for schools that exhibit low levels of exposure and low levels of bias, such as West Charlotte High or LeFlore Magnet (shown in the top left quadrant of Fig. 5a), increasing socioeconomic integration (exposure) is a necessary first step to increasing EC.

The preceding analysis focuses on how to maximize EC from the perspective of a given student with low SES (that is, how to increase the likelihood that they form cross-SES friendships within a given school). However, from a social perspective, it may be more relevant to consider a given school’s contribution to the total number of cross-SES friendships in society. To see how these concepts differ, consider Phillips Exeter Academy, an elite private school in New Hampshire where almost 80% of students come from families with above-median SES (exposure is high) and friending bias is low (below zero). Given these conditions, Phillips Exeter students with low SES tend to form many friendships with their high-SES classmates and have a high EC. However, because students with low SES make up only a small share of Phillips Exeter’s students, the total number of cross-SES connections that Phillips Exeter generates is relatively small. If Phillips Exeter were to enrol more students with low SES (and fewer with high SES), it could increase its total contribution to connectedness despite reducing EC for current low-SES students (as they would be exposed to fewer high-SES peers).

We measure each school’s total contribution to EC (TCEC) as the product of the share of low-SES students and the average EC among low-SES students in that school (Methods: ‘Total contribution to connectedness’). TCEC measures how many friendships a school creates between students with high and low SES, holding fixed total enrolment and the total number of friends that students make across schools. Reducing friending bias at a school (all else equal) always increases the total number of friendships between students with low and high SES. However, increasing the share of high-SES students has non-monotonic effects on TCEC. Schools that have very few high-SES students offer few opportunities for their low-SES students to meet high-SES peers and therefore contribute little to overall economic connectedness. Conversely, schools that have predominantly high-SES students, such as Phillips Exeter or the Dalton School in New York City, provide many high-SES connections to the low-SES students that they do enrol, but offer those opportunities to relatively few low-SES students and therefore also have low TCEC.

Owing to these competing forces, when holding friending bias fixed, an above-median-SES share of 50% (that is, achieving perfect socioeconomic integration) maximizes the total number of cross-class connections at a school. Schools that have low friending bias and near-equal representation of students with below- and above-median parental SES—such as Lane Technical in Fig. 5a—contribute the most to total EC in an accounting sense. More generally, the direction in which one must shift exposure to increase the total number of cross-SES links differs on the basis of a school’s initial share of high-SES students. By contrast, reducing friending bias always increases EC for a given low-SES student as well as TCEC.

Furthermore, increasing the share of high-SES students in one school necessarily requires reducing the share of high-SES students in at least one other school, as the total number of students with above-median SES is fixed. As a result, increasing high-SES shares even at schools where high-SES shares are below 50% can have ambiguous effects on EC in society as a whole. If the high-SES students who join a given school A otherwise would have attended school B where they would have connected with more low-SES peers, overall EC in society could fall even though TCEC at school A would rise. Thus, one must be cognizant of the counterfactual distribution of SES across schools when evaluating the effects of increasing exposure. By contrast, efforts to reduce friending bias in a given school do not generally have direct implications for connectedness at other schools.

In summary, for schools that already have diverse student bodies (that is, schools that have a balanced socioeconomic representation) but high levels of friending bias, initiatives to identify and address institutional factors contributing to friending bias may be the most fruitful path to increasing their total contributions to connectedness. For schools that currently have less diverse student bodies, it may be valuable to increase diversity in a manner that takes account of which schools the new students would otherwise have attended.

## Exposure and friending bias by college

Figure 5b replicates Fig. 5a for colleges, again using parental SES. We see analogous heterogeneity in exposure and friending bias across colleges, with similar implications. For example, Yale University exhibits relatively low friending bias and has a large high-SES share, resulting in high levels of EC for its low-SES students. However, because students with low SES make up only a small share of the student body, Yale, similar to many other elite private colleges, creates relatively few cross-SES connections (it has low TCEC).

Among colleges with more socioeconomically diverse student bodies, such as Wayne State and Howard, there is again considerable variation in connectedness that results from differences in friending bias. Similar to high schools, friending bias tends to increase with a college’s size and with the degree of racial diversity of the student body (Supplementary Fig. 3). In a different vein, ethnographic evidence suggests that many colleges that exhibit high levels of bias—such as the University of Alabama, Syracuse University, or the University of Mississippi—feature significant Greek life, where the high costs of fraternity and sorority dues may generate friending bias on campus^36^. Similarly, community colleges without a substantial residential student population (for example, the City College of San Francisco or San Diego City College) tend to exhibit high levels of friending bias. Systematically evaluating these and other hypotheses using the data constructed here would be a useful direction for further work. For now, these results again suggest that friending bias is at least partly determined by structural factors that could potentially be changed by colleges, much like recent efforts to increase socioeconomic diversity at elite private colleges.

## Effects of integration on connectedness

Having established that there is significant variation across schools and colleges in friending bias and exposure, we now examine whether these estimates are sufficiently reliable to determine what interventions will be most effective at increasing EC in a given school. As a practical illustration, consider policies that seek to increase socioeconomic diversity in a given school district. We examine whether our estimates of average friending bias can be used to reliably identify the schools in which such policies will increase connectedness the most. If estimates of friending bias are perfectly stable, the effect of a change in socioeconomic composition will be well predicted by historical estimates of average friending bias. By contrast, if estimates of bias change over time (for example, due to measurement error or drift), or if the effects of incremental changes in socioeconomic diversity on EC differ substantially from historical averages of friending bias, predictions based on existing observational data may not provide reliable forecasts. It is therefore an empirical question whether the school-level estimates that we report provide useful information to predict the effects of policy changes. We use two quasi-experimental research designs to identify the causal effects of changes in exposure on connectedness—cross-cohort fluctuations and regression discontinuity—and show that our school-level estimates of average friending bias predict the causal effects of these changes in exposure on economic connectedness.

### Cross-cohort fluctuations

In our first approach, we analyse the effects of fluctuations in the share of students with high SES across cohorts within a high school on students’ friendship patterns. Such fluctuations in cohort composition are largely a consequence of random variation in the student body, as discussed in the ‘Cross-cohort fluctuations’ section of the Methods. Intuitively, we compare low-SES students who attend the same school and examine whether those who happen to be in cohorts that have a larger share of high-SES students tend to have more high-SES friends as a result. To harness more variation across cohorts, we focus here on connections between individuals with parents in the lowest and highest SES quintiles (rather than below- versus above-median SES, as we do in the rest of the paper).

**Fig. 6:**
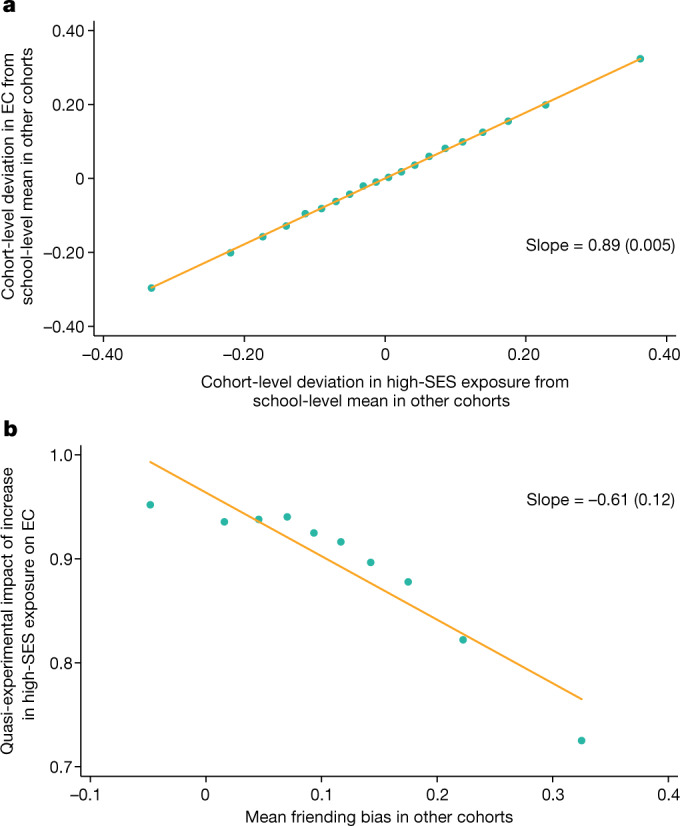
Cross-cohort estimates of the causal effects of socioeconomic integration on EC in high schools. **a**,**b**, Analysis of the causal effect of being assigned to a high school cohort with more high-SES peers on the EC of low-SES students, based on the level of friending bias in the school (Methods: ‘Cross-cohort estimates’). **a**, Cohort-level changes in economic connectedness of low-SES students versus changes in the share of high-SES students. **b**, Causal impacts of high-SES share on economic connectedness of low-SES students, by level of friending bias. We measure EC, exposure and bias in this figure based on parental SES. The sample consists of all of the individuals in our primary analysis sample who were born between 1990 and 2000 whom we can link to parents and match to high schools. We further limit the sample to schools with at least 500 students (pooling all cohorts), at least 100 bottom-quintile-SES students and at least 100 top-quintile-SES students. For each cohort, exposure is defined as five times the fraction of top-quintile-SES students. EC in a cohort is defined as five times the average share of top-quintile-SES friends among bottom-quintile-SES students. Friending bias is defined as the average among bottom-quintile-SES students of one minus the ratio of the share of friends with top-quintile SES to the share of peers with a top-quintile SES in their cohort. In **a**, a binned scatter plot is shown of the cohort-level deviations from school means in EC versus cohort-level deviations from school means in exposure. The cohort-level deviations are constructed as the mean for the relevant cohort *c* in a given school minus the mean for all other cohorts in the same school, weighting by the number of students with bottom-quintile SES in each cohort. The binned scatter plot is constructed by dividing the cohort-level deviations in exposure into 20 equally sized bins and plotting the mean deviation in EC versus the mean deviation in exposure within each bin. We also report a slope estimated using a linear regression, with standard error clustered by high school in parentheses. To construct the plot in **b**, we first divide school × cohort cells into deciles based on the mean level of friending bias for all other cohorts in the same school. We then estimate regressions analogous to that in **a** using the school × cohort cells in each of the ten deciles separately. Finally, we plot the slopes from the ten regressions against the mean level of friending bias (leaving out the focal cohort) in each decile.

Figure 6a presents a binned scatter plot of changes in EC for low-SES students across cohorts within a school versus cross-cohort changes in high-SES exposure (Methods: ‘Cross-cohort fluctuations’). In this analysis, we focus on measuring within-cohort EC and exposure—that is, the shares of high-SES friends and peers that low-SES students have within only their own cohorts in their high schools. The strong positive relationship demonstrates that, within a given school, students in cohorts that happen to have more high-SES students have significantly more high-SES friends in their cohorts on average. Thus, greater high-SES exposure translates to a significantly greater number of high-SES friendships on average, showing that socioeconomic integration can be a powerful tool for increasing cross-class interaction.

The slope of 0.89 in Fig. 6a implies marginal friending bias of 0.11: a 10 percentage point increase in the share of high-SES peers in a given cohort leads to an 8.9 percentage point increase in the share of high-SES friends among low-SES students in that cohort on average. The corresponding cross-sectional mean of bottom to top parental-SES-quintile friending bias is also 11%. Thus, an incremental change in socioeconomic integration has a similar causal effect on connectedness to what one would predict on the basis of the average level of friending bias in the observational data.

Next, we consider how the relationship in Fig. 6a varies across schools that have different levels of friending bias. We estimate a regression analogous to that shown in Fig. 6a separately for school-cohort cells in each decile of the friending bias distribution (estimating friending bias based on data for other cohorts in the same school). Figure 6b plots the estimated regression coefficients in each decile against the level of friending bias in that decile. There is a strong negative relationship, showing that an increase in high-SES exposure produces fewer cross-SES friendships in schools that exhibit higher friending bias. The slope of the relationship in Fig. 6b is −0.61, implying that a 1 percentage point increase in mean friending bias in other cohorts translates to a 0.61 percentage point reduction in the effect of exposure on EC.

This coefficient may be below 1 for two different reasons. First, sampling error in our estimates of friending bias leads to imperfect predictions of friending bias in a given cohort. Second, the average level of friending bias observed in a school may not correspond to the bias associated with befriending an incremental high-SES student in a cohort. To distinguish between these explanations, note that in the sample used for the quasi-experimental analysis in Fig. 6, a 1% increase in mean friending bias in other cohorts is associated with a 0.67 percentage point increase in friending bias in a given cohort *c* on average. Correcting for this degree of attenuation bias, the implied impact of a 1% increase in average friending bias in a given cohort is a 0.61/0.67 = 0.91 percentage point reduction in the impact of an incremental change in exposure on EC. Thus, fluctuations in exposure translate to cross-SES friendships at close to the rate that one would expect given the average friending bias in a given cohort. This finding supports the use of average observed friending bias in a school to predict the effects of incremental changes in exposure on EC, in particular after accounting for sampling error in friending bias.

### Regression discontinuity

If students with high parental SES move into certain school districts over time and those districts also exhibit secular trends in cross-SES friendships (for example, due to changes in friending bias) for other unrelated reasons, the cross-cohort comparisons above may yield biased estimates of the causal effect of exposure on EC. To address such concerns, we now turn to a second approach that leverages the fact that most states use cut-offs based on birth dates to determine when students begin school; for example, in Texas, students who turn five years old on or before 1 September begin Kindergarten that year, whereas those who turn five on or after 2 September begin school the next year (Supplementary Table 2). We use these cut-offs to implement a regression discontinuity design, comparing EC for low-SES individuals who happen to fall on different sides of the entry cut-off (for example, are born on 1 September versus 2 September) and are therefore exposed to high school peer groups that differ in their share of high-SES students. See the ‘Regression discontinuity’ section of the Methods for a discussion of the identification assumptions underlying this design and further details.

**Extended Data Fig. 4:**
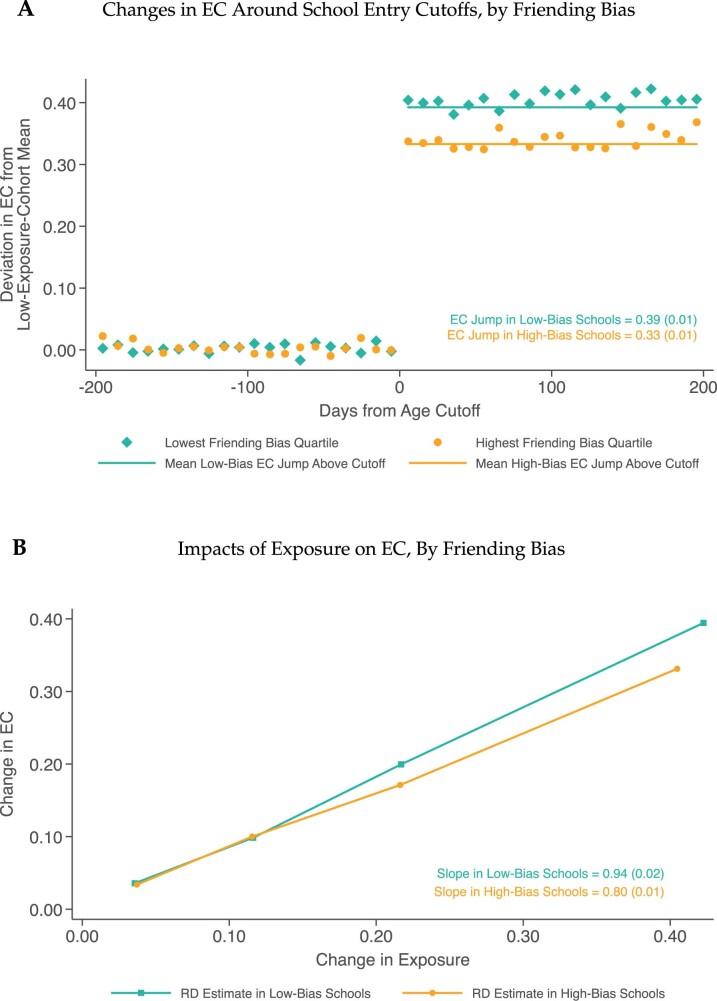
Causal Effects of Socioeconomic Integration on Economic Connectedness in High Schools: Regression-Discontinuity Estimates. This figure analyses the causal effects of being assigned to a high school cohort with more high-SES peers on economic connectedness (EC) using a regression discontinuity design, separately for schools with low versus high levels of friending bias (Methods: ‘Regression Discontinuity Estimates’). The sample consists of all individuals in our analysis sample born between 1990 and 2000 whom we can link to their parents and match to high schools. Panel A shows the reduced-form impacts of jumps in the share of high-SES students on EC, separately by the level of friending bias. To construct Panel A, we first calculate the absolute values of changes in the fraction of students with top-quintile-parental-SES ("high-SES”) across all consecutive high-school cohort pairs. We then restrict the sample to cohort pairs in the top quartile of this distribution of exposure changes, and order cohorts so that the cohort with the lower share of high-SES students is the first of the two cohorts. We define EC for a given student as five times the share of top-quintile-parental-SES friends in their cohort. We then calculate means of EC for all bottom-quintile-parental-SES ("low-SES") students in a cohort pair, pooling students by their age distance in days from the cohort age cutoff (subtracting the mean EC among low-SES students in the cohort). We do this separately for schools in the top and bottom quartile of the distribution of friending bias, calculated as the average friending bias of low-SES individuals in the same high school over all cohorts excluding the own and adjacent cohorts. We report regression discontinuity (RD) estimates (with standard errors in parentheses) of the jump in average EC at the cutoff estimated using a linear regression with a bandwidth of 200 days; the solid lines plot the magnitudes of these jumps (Methods: ‘Regression Discontinuity Estimates’). In Panel B, we plot RD estimates of the jump in EC (estimated as in Panel A) for each of the four quartiles of changes in exposure across cohorts against the mean change in (normalized) high-SES exposure in that exposure-jump quartile. We again plot these estimates separately for schools in the top and bottom quartile of leave-out friending bias.

We begin by focusing on pairs of adjacent cohorts in which the magnitude of the jump in the share of high-parental-SES students is large, that is, lies in the top quartile of the distribution of changes in high-SES shares. In Extended Data Fig. 4a, we examine how these jumps in exposure to peers with high parental SES affect within-cohort economic connectedness. We examine these effects separately in schools with low (bottom quartile) versus high (top quartile) friending bias. The share of friends with high parental SES jumps to the right of the school entry cut-off in both sets of schools, showing that exposure to more high-SES peers in one’s cohort (that is, greater exposure) leads students to form more high-SES friendships within their school cohorts. However, the magnitude of the jump in high-SES friendships caused by this increased exposure is 0.06 units greater in schools with low friending bias compared with in schools with high friending bias. This difference is similar to the observed difference in average friending bias between schools classified (on the basis of data from other cohorts) to be in the bottom versus top quartile of friending bias, again demonstrating that the observed average friending bias (adjusted for measurement error) predicts the effect of incremental changes in exposure on EC accurately.

In Extended Data Fig. 4b, we extend this approach to look beyond cohort pairs with large fluctuations in high-SES shares. We plot regression discontinuity estimates of changes in within-cohort EC versus changes in exposure for each of the four quartiles of changes in high-SES exposure across cohorts, separately for schools in the bottom and top friending bias quartiles. The right-most points in this figure match the regression discontinuity estimates reported in Extended Data Fig. 4a. Low-SES students’ shares of high-SES friends increase linearly with their exposure to high-SES peers across the distribution of exposure changes. The slope of the line is steeper in schools with low friending bias, showing that greater high-SES exposure translates to greater cross-SES friendships when friending bias is low.

We conclude that our school-specific observational estimates of friending bias are sufficiently stable and reliable for predicting the causal effects of changes in exposure on EC out of sample, and can therefore inform where efforts to reduce friending bias versus increase exposure are likely to be most valuable.

## Discussion

The extent to which individuals interact across class lines depends on both exposure (the socioeconomic composition of the groups to which people belong) and friending bias (the rate at which cross-SES friendships are formed conditional on exposure). To date, there have been extensive policy efforts on the exposure dimension, such as busing programs aimed at integrating schools; zoning and affordable housing policies aimed at integrating neighbourhoods; and college admissions reforms to boost diversity on campuses. Such interventions to increase integration can increase cross-SES interaction substantially. However, even if all such groups were perfectly integrated by socioeconomic status, half of the social disconnection between people with low and high socioeconomic status would persist because of friending bias within groups.

Our analysis suggests that friending bias, like exposure, is shaped by social structures and institutions and can therefore be influenced by policy changes. Although interventions to reduce friending bias have been studied less extensively^37,38^, there are several recent initiatives that seek to reduce friending bias. We conclude by discussing some examples to illustrate the types of interventions that could be studied and scaled going forward.

(1) Changes in group size and tracking. As discussed above, Berkeley High School (BHS) has historically been socioeconomically diverse, but has had high levels of friending bias. Those familiar with the school were aware of this issue and pointed to tracking as a source of substantial within-school segregation. For example, Kim^39^ writes: “BHS’s population of more than 3,000 students is currently split into five learning communities, each meant to provide its own focus and curriculum....causing implicit segregation, resulting in student learning communities with separated concentrations of white students and students of color”. In an attempt to overcome this within-school segregation and to reduce the associated friending bias, in 2018 BHS began assigning students to small, intentionally diverse ‘houses’ or ‘hives’ in ninth grade. Such attention to the way in which students are tracked to different classes within schools and the size of the groups in which they participate outside class may be helpful in reducing friending bias more broadly.

(2) Restructuring of space and urban planning. Lake Highlands High School in Texas is another school in which we observed high levels of friending bias (Fig. 5a). In this case, administrators and students at Lake Highlands High identified the architecture of the building as an impediment to cross-SES interaction: “At Lake Highlands High, the duplicated rooms—cafeterias, libraries, science labs—led to unintentional student segregation,” such that “students clustered in one of three lunchrooms depending on their social group or the options for low-cost and free lunch^40^”. The school recently attempted to reduce this source of friending bias through a large-scale construction project that created a single cafeteria and more spaces for all students to interact. One of the architects described the project’s goals as follows: “shrink income-based inequalities in education by designing schools that improve the way students learn and socialize”, noting that “though students may still split into their own cliques [...], they’ll have more opportunities to cross paths and interact with peers from other social groups”. Architecture and urban planning could have a role in reducing friending bias outside schools as well. Examples include social infrastructure, such as public libraries, to build social bonds across groups^41^; the effects of public parks on social interactions^42^; and the impacts of public transit on the interactions between people living in different neighbourhoods^43^.

(3) New domains for interaction. Another approach to reducing friending bias could be to create new programs and venues for cross-SES interaction. For example, the Boston gym Inner City Weightlifting (ICW) began a program to increase cross-SES connections by recruiting personal trainers from lower-SES backgrounds to coach their more affluent clients. The founder J. Feinman described the objective as follows: “At ICW, through our career track in personal training, we help create economic mobility for people in our program as they begin earning $20–$60 per hour training clients from opposite socioeconomic backgrounds. More importantly, this flips power dynamics, bridges social capital, and creates a genuine form of inclusion that disrupts the system of segregation, isolation, and racism that leads to the streets. The people in our program gain access to new networks and opportunities, while our clients gain new insights and perspectives into complex social challenges^44^”. Feinman notes that the program appears to be a success: “Along the way, something unexpected happened. We had our paying clients—people paying our student trainers—visiting our students in jail when things went wrong. They were showing up in court to be a support. They started offering job opportunities to our students outside of the gym, and they paid for the children of our students to go to summer camp with their own children^45^”. More generally, creating new programs and venues for cross-SES interaction (for example, through peer mentoring programs or internship programs) could help to reduce friending bias.

The school-, college- and ZIP-code-level data on exposure and friending bias made publicly available through this project can help to determine whether interventions to reduce friending bias or efforts to increase socioeconomic diversity are likely to be most valuable for increasing economic connectedness. Going forward, the methods and data developed here can be used to evaluate the causal effects of interventions such as those described above. By studying changes in exposure and friending bias over time, researchers and policy-makers can learn from places in which progress is being made and provide assistance to communities seeking to improve economic connectedness—the form of social capital that is most strongly associated with economic mobility.

## Methods

### Sample construction

This section describes the methods used to generate the data analysed in this paper. A server-side analysis script was designed to automatically process the raw data, strip the data of personal identifiers, and generate aggregate results, which we analysed to produce the conclusions in this paper. The script then promptly deleted the raw data generated for this project (see the ‘Privacy and ethics’ section).

We start from the analysis sample constructed in our companion paper^7^: users aged between 25 and 44 years as of 28 May 2022, who reside in the United States, were active on the Facebook platform at least once in the previous 30 days, have at least 100 US-based Facebook friends and have a non-missing predicted ZIP code. We then restrict attention to individuals for whom we can allocate at least one friendship to the group in which it was formed (using the approach described below). The resulting sample consists of 70.3 million Facebook users, corresponding to 82% of the US population between the ages 25 and 44 years based on the American Community Survey (ACS).

We do not link any external individual-level information to the Facebook data. However, the project uses various publicly available sources of aggregate statistics to supplement the analysis, such as data on median incomes by block group from the 2014–2018 ACS; school-level variables from the National Center for Education Statistics (NCES) and the Civil Rights Data Collection (CRDC); and various college-level statistics from the Integrated Postsecondary Education Data System (IPEDS) and ref. ^46^. We describe those data in detail in Supplementary Information A.

### Variable definitions

We construct the following sets of variables for each person in our analysis sample; the first four variables are identical to those used in our companion paper^7^, while the fifth is new to this paper.

#### Friendship links

The data contain information on all friendship links between Facebook users. Facebook friendship links need to be confirmed by both parties, and most links are between individuals who have interacted in person^47^. As a result, the Facebook friendship network can be interpreted as providing data on people’s real-world friends and acquaintances rather than purely online connections.

#### Locations

Every individual in our analysis sample is assigned a ZIP code and county based on information and activity on Facebook, including the city stated on their Facebook profile as well as device and connection information. Formally, we use 2010 census ZIP code tabulation areas to perform all geographic analyses of ZIP code-level data. We refer to these ZIP code tabulation areas as ZIP codes for simplicity. According to the 2014–2018 ACS, there are 219,214 census block groups, 32,799 ZIP codes and 3,220 counties, with average populations of 1,488, 9,948 and 101,332 in each respective geographical designation.

#### Socioeconomic status

Social scientists have measured socioeconomic status (SES) using many different variables, ranging from income and wealth to educational attainment, occupation, family background, neighbourhood and consumption^21^. To capture these varied definitions, we construct a model that generates a composite measure of SES for working-age adults (individuals between the ages of 25 and 64 years) that combines various characteristics (see the ‘Privacy and ethics’ section for a discussion of how user privacy was protected during this project). We construct our baseline SES measure in three steps (see Supplementary Information B.1 of our companion paper^7^ for details). First, for Facebook users who have Location History (LH) settings enabled, we compute the median household income in their Census block groups. Location history is an opt-in setting for Facebook accounts that enables the collection and storage of location signals provided by a device’s operating system while the app is running. We observe census block groups from individuals in the location history subsample; by contrast, we can assign individuals who do not have location history enabled only to ZIP codes. If an individual subsequently opts out of location history, their previously stored location signals are not retained.

Second, we estimate a gradient-boosted regression tree to predict these median block group household incomes using variables observed for all individuals in our sample, such as age, gender, language, relationship status, location information (ZIP code), college, donations, phone model price and mobile carrier, usage of Facebook on the web (rather than a mobile device) and other variables related to Facebook usage. We use this model to generate SES predictions for all of the individuals in our sample.

Finally, individuals (including the location history users in the training sample) are assigned percentile ranks in the national SES distribution on the basis of their predicted SES relative to others in the same birth cohort.

We do not use any information from an individual’s friends to predict their SES, ensuring that errors in the SES predictions are not correlated across friends, which would bias our estimates of homophily by SES. We also do not use direct information on individuals’ incomes or wealth, as we do not observe these variables at the individual level in our data; however, we show below that our measures of SES are highly correlated with measures of income across subgroups. Note that the algorithm described above is one of many potential ways of combining a set of underlying proxies for SES into a single measure; other methods discussed in our companion paper^7^ yield very similar results.

#### Parental SES

We link individuals in our primary analysis sample (that is, those aged 25–44) to their parents (who may not be in the analysis sample themselves) to construct measures of family socioeconomic status during childhood. To link individuals to their parents, we use self-reported familial ties, a hash of user last names, and public user-generated wall posts and major life events^7^. We then use the SES of parents, constructed using the algorithm described above, to assign parental SES to individuals. We are able to assign parental SES ranks for 31% of the primary analysis sample.

#### Groups where friends are made

We assign friendships to the groups in which they were made by focusing on six settings (group types) that we can identify reliably in our data: high schools, colleges, employers, neighbourhoods (ZIP codes), faith-based (religious) groups and recreational groups. These settings span the most common places in which users make friends, excluding family^19,20^.

We first use self-reported data (for colleges, employers and high schools), liked pages of places of worship (religious groups) and group membership (recreational and religious groups) to assign individuals to at most one group in each setting (Supplementary Information B.1). For some people who do not report a high school, we use data on their friendship networks to impute their high school. For the small set of individuals who are members of multiple groups within a setting (for example, 3.3% of users report multiple high schools conditional on being assigned a high school), we select the group in which the user has the largest number of friends. The quality of our group assignments appears to be high based on comparisons to external statistics. For example, our estimates of the share of high-SES households in each ZIP code, high school and college have correlations above 0.85 with corresponding statistics drawn from publicly available administrative datasets (Extended Data Table 2).

We then assign friendship links to groups on the basis of shared group membership, as described in Supplementary Information B.2. For example, if an individual and one of their friends are part of the same neighbourhood, they are identified as neighbourhood friends. In cases with shared group membership across multiple settings—for example, when two friends are members of both the same recreational group and the same workplace—the friendship link is counted in all relevant settings. We are able to allocate about 30% of friendship links to at least one setting. The remaining friends either could not be connected to a group due to missing data (for example, missing data on the workplace of the users or friends) or were made outside the settings we consider. Note that this research did not involve inferences about an individual’s religion; instead, it is focused on whether friendships were formed in a faith-based (religious) group. 

### Benchmarking

Extended Data Table 3a shows summary statistics for the primary analysis sample used in this paper (as of 28 May 2022) and, for comparison, for those between ages 25–44 years in the 2014–2018 ACS. As discussed in our companion paper^7^, the Facebook sample is quite similar to the full population in terms of age, gender and language. The companion paper^7^ further demonstrates that the Facebook sample is broadly representative of the US population geographically and that the SES measures used in our analysis below are well correlated with publicly available statistics and yield estimates of homophily by SES and intergenerational mobility that match external estimates from nationally representative datasets.

When analysing interventions to increase EC in high schools and colleges, we focus on the subsample of individuals who can be assigned a high school or college and who can be linked to parents with an SES prediction (to measure connectedness by parental SES). Extended Data Table 3b presents summary statistics for the subsample of 19.4 million users who can be assigned parental SES and high school, who constitute 28% of the full analysis sample. The characteristics of this subsample are broadly similar to those of the full sample.

**Extended Data Fig. 5:**
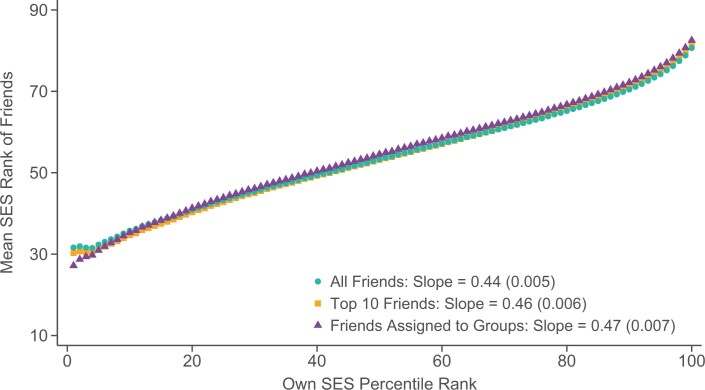
Relationship Between Friends’ Socioeconomic Status and Own Socioeconomic Status. This figure plots the mean socioeconomic status (SES) percentile rank of individuals’ friends against their own SES percentile rank. The series in green circles is calculated using the entire friendship network for each individual. The series in orange squares is constructed using each individual’s ten closest friends, based on the frequency of public interactions such as likes, tags, wall posts, and comments. The green and orange series are identical to those in Fig. 1 of Chetty *et al*.^7^. The series in purple triangles replicates the series in green circles using the 30% of friendships that we are able to assign to a group, the primary analysis sample used in this paper. For each series, we report slopes estimated from a linear regression on the plotted points, with heteroskedasticity-robust standard errors in parentheses.

In this paper, we focus on the 30% of friendships that can be assigned to groups in which people interact, which is necessary to identify exposure and friending bias. We find that within the subsample of friendships that can be assigned to groups, homophily is similar to that observed in the full sample of friendships (Extended Data Fig. 5). Moreover, at the individual level, a person’s share of high-SES friends in the subsample of friends assigned to a group has a correlation of more than 0.90 with their share of high-SES friends overall. Furthermore, to address potential concerns about bias from under-reporting of groups, we developed a procedure to correct for under-reporting of group memberships using external statistics on group membership rates (Supplementary Information B.3). In this expanded sample, which accounts for 44% of friendships, our conclusions remain similar (Supplementary Fig. 4).

On the basis of this analysis, we conclude that the subsample of friendships that we analysed here is reasonably representative of the broader set of friendships that people make on Facebook and in the population in general.

### Decomposing EC

Following our companion paper^7^, we define individuals’ economic connectedness as the extent to which they are friends with high-SES individuals. Formally, let *f*_*H*,*i*_ denote individual *i*’s share of high-SES friends and let *w*_*H*_ = 0.5 represent the share of above-median-SES individuals in the population. We define person *i*’s individual economic connectedness (IEC) to high-SES individuals as:

1 $${{\rm{IEC}}}_{H,i}\equiv \frac{{f}_{H,i}}{{w}_{H}}.$$

If IEC_*H*,*i*_ > 1, individual *i* has more high-SES friends than one would expect if friendships were made at random and low- and high-SES people made an equal number of friends; conversely, IEC_*H*,*i*_ < 1 means that *i* has fewer high-SES friends than one would expect under random friending.

To decompose *IEC*_*H*,*i*_ into exposure and friending bias, let *ϕ*_*i*,*g*_ denote the fraction of friends that individual *i* makes in group *g* (out of all friends of *i* that can be assigned to groups) and let *G* denote the set of all available groups, so that ∑_*g*∈*G*_*ϕ*_*i*,*g*_ = 1 for each individual. Here, a group *g* represents a specific school, college, recreational group, and so on, to which an individual can belong. Individuals’ friending shares *ϕ*_*i*,*g*_ are positive or 0 in the specific groups to which they belong and are 0 for all other groups. Let *w*_*H*,*g*_ denote the fraction of members of group *g* who have high SES and *f*_*H*,*i*,*g*_ the fraction of friends individual *i* makes in group *g* who have high SES (see Supplementary Information B.4 for a discussion of how we define *f*_*H*,*i*,*g*_ when *ϕ*_*i*,*g*_ = 0).

We can express each individual’s connectedness to high-SES individuals as the product of three components, summed across groups:

2 $$\begin{array}{cll}{{\rm{IEC}}}_{H,i} & = & \frac{{f}_{H,i}}{{w}_{H}}=\sum _{g\in G}\left[{\varphi }_{i,g}\times \frac{{f}_{H,i,g}}{{w}_{H}}\right]=\sum _{g\in G}\left[{\varphi }_{i,g}\times \frac{{w}_{H,g}}{{w}_{H}}\times \frac{{f}_{H,i,g}}{{w}_{H,g}}\right]\\  & = & \sum _{g\in G}[{\varphi }_{i,g}\times {{\rm{Exposure}}}_{H,g}\times (1-{\rm{Friending}}\,{{\rm{bias}}}_{H,i,g})],\end{array}$$

where

3 $${{\rm{Exposure}}}_{H,g}\equiv \frac{{w}_{H,g}}{{w}_{H}}$$

is the normalized fraction of high-SES individuals in group *g*. Exposure is below 1 for groups that have a below-average share of high-SES individuals and above 1 for groups that have an above-average share of high-SES individuals. The final term,

4 $${\rm{Friending}}\,{{\rm{bias}}}_{H,i,g}\equiv 1-\frac{{f}_{H,i,g}}{{w}_{H,g}},$$

measures the deviation from uniformly random friending conditional on exposure.

If friendships were formed at random and if people with high and low SES made the same number of friends, then *f*_*H*,*i*,*g*_ = *w*_*H*,*g*_ and friending bias would be equal to 0. In practice, high-SES individuals make 25.4% more friends than low-SES individuals do on average^7^. Maintaining this difference in the number of total friends, in a setting with no homophily by SES (that is, a setting in which low- and high-SES individuals have the same probability of befriending a given high-SES person), friending bias would be negative. In particular, if high-SES individuals have *x*_*g*_ > 1 times as many friends as low-SES individuals in group *g* but there is no homophily by SES,

5 $${\rm{Friending}}\,{{\rm{bias}}}_{H,i,g}=\frac{1-{x}_{g}}{1+\frac{{w}_{H,g}}{1-{w}_{H,g}}{x}_{g}} < 0.$$

In a group that is representative of the overall population—where *x*_*g*_ = 1.254 and *w*_*H*,*g*_ = 0.5—friending bias would be −0.11, and low-SES individuals would have 11% more high-SES friends than the share of high-SES individuals in the group. Relative to this benchmark, a positive value of friending bias constitutes a substantial departure from a society that does not exhibit homophily by SES, as it means that fewer than half of the friends of low-SES individuals have high SES even though high-SES individuals form more friendships on average.

#### Decompositions by SES

In the ‘Decomposing connectedness by SES’ section, we quantify how much of the difference in the share of high-SES friends between people with low and high SES is due to differences in friending shares across settings, differences in exposure and differences in friending bias. Since the parameters in equation (2) vary across individuals and groups even at a given SES level, we take a representative agent approach to decompose the relative contributions of these three factors on average. In particular, we consider a representative low-SES agent and high-SES agent who have setting-level friending shares, exposure rates and friending bias levels that match the means for low- and high-SES people in the population, respectively (Figs. 1 and 2 and Extended Data Table 4). To conduct the decomposition, we first calculate IECs for the representative low-SES and high-SES agents using these mean values and a formula analogous to equation (2) (Supplementary Information B.5). We then sequentially set each of the parameters for the low-SES agent to match the values for the high-SES agent, enabling us to determine how much of the difference in the representative-agent IEC across the two SES groups is explained by each of the three factors. We refer to the representative low-SES and high-SES agents as the average low- and high-SES persons in the main text.

Because equation (2) is not additive, the share of the total difference attributed to friending bias versus exposure depends on the order in which we conduct each of the steps of the decomposition exercise. For the decomposition by SES discussed in the main text, we equated rates of exposure before rates of friending bias, effectively examining what the effects of socioeconomic integration would be absent any changes in friending bias. If we instead equate rates of friending bias before rates of exposure (Fig. 3a, fourth bar), 34% of the EC gap would be explained by friending bias and 54% by exposure. Lower friending bias and greater exposure are complements, so a factor has the largest effect if it is equated last. Put differently, reducing friending bias leads to more high-SES friends when exposure is higher (and vice versa).

#### Decompositions across areas

We use a similar approach to analyse why the EC among low-SES people varies geographically. We begin by calculating, for every ZIP code, average friending shares by setting, average friending bias (Friending bias_*H*,*L*,*a*_) and average high-SES exposure (Exposure_*H*,*L*,*a*_) of low-SES individuals living in that area (see Supplementary Information B.5 for formal definitions). We then consider representative agents with friending shares across settings and setting-specific levels of exposure and friending bias that match the mean values of these parameters for low-SES individuals living in ZIP codes in the bottom versus top quintiles of the ZIP-code-level EC distribution. We then sequentially set each of the parameters for the representative bottom-ZIP-quintile agent to match the values for the representative top-ZIP-quintile agent (Supplementary Information B.5).

### Exposure, bias and upward income mobility

In Table 1, we analyse the relationship between upward income mobility and EC across counties and ZIP codes, comparing the effects of exposure versus friending bias. Starting from the area-level mean values for exposure and friending bias among low-SES individuals (see the ‘Decompositions across areas’ section), we first create a recomposed measure of EC as the product of the average values of exposure and one minus friending bias in each area *a*:

6 $${{\rm{EC}}}_{H,L,a}^{{\rm{rec}}}={{\rm{Exposure}}}_{H,L,a}\times (1-{\rm{Friending}}\,{{\rm{bias}}}_{H,L,a}).$$

Note that ${{\rm{EC}}}_{H,L,a}^{{\rm{rec}}}$ differs from the measures of area-level EC analysed in our companion paper^7^ because (1) the measure here is based only on the subset friendships that can be assigned to groups and (2) the product of the area-level averages does not take into account the covariances between friend shares, exposure and bias at the individual level. Nevertheless, the two measures of area-level EC have a population-weighted correlation of above 0.95 across both counties and ZIP codes.

As EC is proportional to the product of exposure and one minus friending bias, we use a log transformation of equation (6) to obtain an additive specification:

7 $$ln({{\rm{EC}}}_{H,L,a}^{{\rm{rec}}})=ln({{\rm{Exposure}}}_{H,L,a})+ln(1-{\rm{Friending}}\,{{\rm{bias}}}_{H,L,a}).$$

We then regress the log of the upward income mobility at the county and ZIP code level on these log-transformed measures of EC (columns 1, 3 and 5 of Table 1) or exposure and friending bias (columns 2, 4 and 6 of Table 1), weighting by the below-median-SES population. In column 7 of Table 1, we define the dependent variable as the log of the causal effect of a county on upward mobility, which we calculate as the average level of upward mobility overall in the United States plus 20 times Chetty and Hendren’s^32^ estimate of the annual causal exposure effect of growing up in that county.

### High school estimates

For both high-school- and college-level estimates of friending bias and exposure using own SES, we focus on the 1986–1996 birth cohorts (measuring SES in 2022, between the ages of 26–36 years). For estimates based on parental SES, we focus on individuals in the 1990–2000 birth cohorts. We focus on more recent birth cohorts for parental SES to maximize the share of individuals we can link to their parents and to measure parental SES (in 2022) before many parents begin to retire. For the 1990–2000 cohorts, we are able to link 46% of individuals assigned to high schools to parents with a non-missing SES rank. We pool data over several cohorts to obtain more precise estimates. School-level estimates of EC are generally stable over time; for example, across schools, EC in the 1978–1982 birth cohorts has a correlation of 0.87 with EC in the 1993–1997 cohorts (Supplementary Fig. 5).

To estimate the reliabilities of exposure and bias for high schools, we first randomly split the population of each high school into two subpopulations, and compute exposure and bias on the subgraphs formed by these two subpopulations. We then take a weighted correlation of these exposure or bias statistics across the split samples, weighting by the number of low-SES students in the school. To adjust for the fact that the estimates are naturally more noisy when estimated on only half of the sample, as opposed to the full sample that we actually use to construct our baseline estimates, we divide the raw split-sample correlation coefficient by the ratio of the (weighted) full-sample variance of EC across schools to the (weighted) split-sample variance of EC across schools.

Individuals with high parental SES make about 22% more friends in high school than individuals with low parental SES^7^. Thus, applying equation (5), we would expect friending bias of −0.10 in a school in which 50% of students have high parental SES and friendships exhibit no homophily, but high-parental-SES students continue to make 22% more friends than  low-parental-SES students.

### Total contribution to connectedness

We define each school *g*’s total contribution to economic connectedness (TCEC) as:

$${{\rm{T}}{\rm{C}}{\rm{E}}{\rm{C}}}_{g}=(1-{{\rm{E}}{\rm{x}}{\rm{p}}{\rm{o}}{\rm{s}}{\rm{u}}{\rm{r}}{\rm{e}}}_{g})\times {{\rm{E}}{\rm{x}}{\rm{p}}{\rm{o}}{\rm{s}}{\rm{u}}{\rm{r}}{\rm{e}}}_{g}\times (1-{\rm{F}}{\rm{r}}{\rm{i}}{\rm{e}}{\rm{n}}{\rm{d}}{\rm{i}}{\rm{n}}{\rm{g}}\,{{\rm{b}}{\rm{i}}{\rm{a}}{\rm{s}}}_{g}),$$

where Exposure_*g*_ and Friending bias_*g*_ are the average high-SES exposure and friending bias of low-SES students in school *g*. In this equation, Exposure_*g*_ × (1 − Friending bias_*g*_) ≈ EC_*g*_, where EC_*g*_ is the average EC of low-SES students in the school. Note that this equality only holds approximately because of the potential covariance between exposure and bias across cohorts within a school. For similar reasons, (1 − Exposure_*g*_) × EC_*g*_ is only approximately equal to the total number of cross-SES links formed per student. Abstracting from any such covariance between exposure and friending bias, TCEC_*g*_ measures a school’s overall contribution to EC per student.

### Cross-cohort estimates

Under the identification assumption that fluctuations in peer group composition across cohorts are orthogonal to other unobservable determinants of students’ friending choices, fluctuations in the share of high-SES peers across cohorts within a school can be used to identify the causal effect of exposure on EC. Previous work in the peer effects literature has found support for this identification assumption using a variety of balance and placebo tests^48^.

To implement the cross-cohort research design, we begin by assigning each person born between 1990 and 2000 to a high school cohort based on their high school and birth date. We use parental SES for this analysis, which—in contrast to children’s own future SES in adulthood—is exogenous to one’s high school peer group. As the design relies on small-sample variation in exposure, we focus on connections between individuals with parents in the lowest and highest SES quintiles (rather than below versus above median SES) to increase variation.

In analogy to a hypothetical experiment that randomly increases the number of high-SES students in a given cohort *c*, let ΔExposure_*sc*_ denote the share of high-SES (top-parental-SES quintile) peers in school *s* in cohort *c* minus the mean share of high-SES peers in school *s* in all other cohorts excluding *c* (divided by the top-quintile population share, 20%). Similarly, let ΔEC_*sc*_ denote the difference in EC between cohort *c* and the mean EC of all other cohorts in the same school. Here, we measure EC within cohorts—that is, the share of high-SES friends that low-SES students make within their cohorts in their high schools. Figure 6a presents a binned scatter plot of ΔEC_*sc*_ versus ΔExposure_*sc*_, pooling all schools.

To construct Fig. 6b, we first divide school-cohort cells into deciles based on the average level of friending bias in all other cohorts in the same school leaving out the focal cohort *c*. We use this leave-out approach to mirror the decision problem of a principal who uses data on existing high school cohorts to estimate school-level bias, and then uses that estimate to predict the effects of future changes in exposure on EC. We estimate a regression analogous to that shown in Fig. 6a separately for cohorts in each decile of the friending bias distribution. Figure 6b shows the estimated regression coefficients in each decile against the level of leave-out friending bias (based on all other cohorts excluding the focal cohort) in that decile.

We use all other cohorts (including future cohorts) to maximize precision when estimating friending bias in Fig. 6, but obtain similar results when we use only prior cohorts to calculate school level friending bias for a given cohort *c*. We also obtain similar estimates when we use first-differences instead of fixed effects (that is, comparing changes in EC and exposure across adjacent cohorts instead of demeaning with respect to all cohorts in the school) and when demeaning EC and exposure in each cohort with respect to the two neighbouring cohorts rather than all cohorts (Supplementary Fig. 6).

### Regression discontinuity estimates

The regression discontinuity design induces quasi-random assignment across adjacent cohorts, and therefore addresses potential biases that could arise in the cross-cohort design from correlated trends in exposure and EC. The identification assumption underlying the regression discontinuity design is that other determinants of friending behaviour do not jump discretely across cohorts in a manner that is correlated with differences in the share of high-SES students across cohorts. We assess the validity of this assumption in Supplementary Information B.6, where we show that observable characteristics do not jump discretely at cohort cut-offs with changes in exposure.

As in the cross-cohort design, we focus on within-cohort friendships between students in the bottom-parental-SES quintile and those in the top-parental-SES quintile. To implement the design, we begin by focusing on pairs of adjacent cohorts in which the magnitude of the jump in top-parental-SES-quintile (high-SES) exposure ∣Δ*E*_*sc*_∣ = Exposure_*sc*_ − Exposure_*s*__,__*c*__−1_ lies in the top quartile of the distribution of ∣Δ*E*_*sc*_∣ across cohort pairs. On average, high-SES exposure jumps by approximately 0.40 units at the entry date cut-off, pooling all cohort pairs in the top quartile of ∣Δ*E*_*sc*_∣.

In Extended Data Fig. 4a, we examine how these jumps in exposure to peers with high parental SES affect individual EC in schools with low (bottom quartile) versus high (top quartile) friending bias. As above, we use leave-out estimates of friending bias for each cohort calculated as the average friending bias in all other cohorts of the same school, excluding the two focal cohorts used in the regression discontinuity analysis. Each series in the figure plots within-cohort EC—the share of high-parental-SES friends that low-parental-SES students have within their high school cohort divided by 0.2, the high-SES population share—versus their date of birth, subtracting the prior cohort mean to isolate changes across cohorts.

For each friending bias quartile, we estimate the magnitude of the jump in EC at the entry cut-off by regressing cohort-specific EC on date of birth, an indicator for being above the entry date cut-off, and the interaction of date of birth with the indicator for being above the entry date cut-off (see Supplementary Information B.6 for the estimating equation). We use a bandwidth of 200 days around either side of the cut-off in this regression; we show the robustness of our estimates to other bandwidths in Supplementary Information B.6.

Extended Data Fig. 4b collects the regression discontinuity estimates obtained from analogous regression specifications for all four quartiles of the distribution of ∣Δ*E*_*sc*_∣. It then plots those estimates versus the mean change in ∣Δ*E*_*sc*_∣ in each quartile, separately for schools in the top versus bottom quartile of friending bias.

### Privacy and ethics

The research is focused on drawing high-level insights about communities and groups of people, rather than individuals. We used a server-side analysis script that was designed to automatically process the raw data, strip the data of personal identifiers, and generate aggregated results, which we analysed to produce the conclusions in this paper. The script then promptly deleted the raw data generated for this project. While we used various publicly available sources of aggregate statistics to supplement our analysis, we do not link any external individual-level information to the Facebook data. All inferences made as part of this research were created and used solely for the purpose of this research and were not used by Meta for any other purpose.

A publicly available dataset, which includes only aggregate statistics on social capital, is available on www.socialcapital.org. We use methods from the differential privacy literature to add noise to these aggregate statistics to protect privacy while maintaining a high level of statistical reliability; see https://www.socialcapital.org for further details on these procedures. The project was approved under Harvard University IRB 17-1692. 

### Reporting summary

Further information on research design is available in the Nature Research Reporting Summary linked to this paper.

## Online content

Any methods, additional references, Nature Research reporting summaries, source data, extended data, supplementary information, acknowledgements, peer review information; details of author contributions and competing interests; and statements of data and code availability are available at 10.1038/s41586-022-04997-3.

### Supplementary information

Supplementary Information

Reporting Summary

## Figures and Tables

**Extended Data Table 1:**
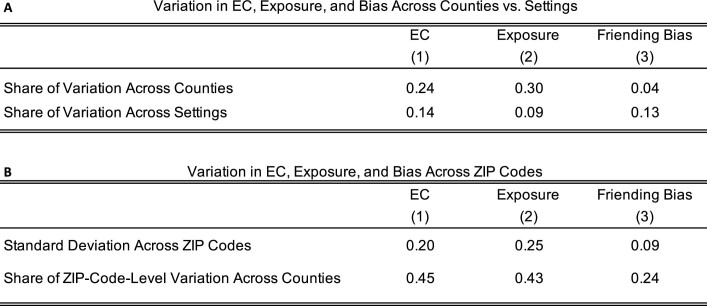
EC, Exposure and Friending Bias: Variation across Areas and Settings

Panel A shows how much of the group-level variation in EC, exposure and friending bias is between counties versus settings. The first column reports the adjusted *R*^2^ from two separate regressions: one where mean economic connectedness (EC) in each group is regressed on county indicators (a group’s county is defined as the modal residential county of individuals assigned to that group), and another where EC is regressed on the six setting indicators. The unit of observation in these regressions is a single group such as a particular high school, neighbourhood, or recreational group. EC is defined as twice the average share of above-median-SES friends in the group among below-median-SES people within each group. Columns 2 and 3 replicate Column 1 with exposure and friending bias as the dependent variables. Exposure is defined as twice the share of above-median-SES individuals in the group. Friending bias is defined as the one minus the mean ratio of the share of above-median-SES friends to the share of above-median-SES peers in the group, averaging over below-median-SES individuals in the group (see Supplementary Information B.5). Panel B focuses on the variation in mean EC, exposure and friending bias at the ZIP-code level. The first row reports the standard deviation of EC, exposure and bias across ZIP codes. The second row shows the adjusted *R*^2^s from regressions of ZIP-code-level means of these three variables on county indicators. To construct ZIP-code-level means, we aggregate individual-level measures of EC, exposure and friending bias as described in the notes to Fig. 4 and Supplementary Information B.5. All statistics are weighted by the number of below-median-SES individuals in each group (Panel A) or ZIP code (Panel B).

**Extended Data Table 2:**
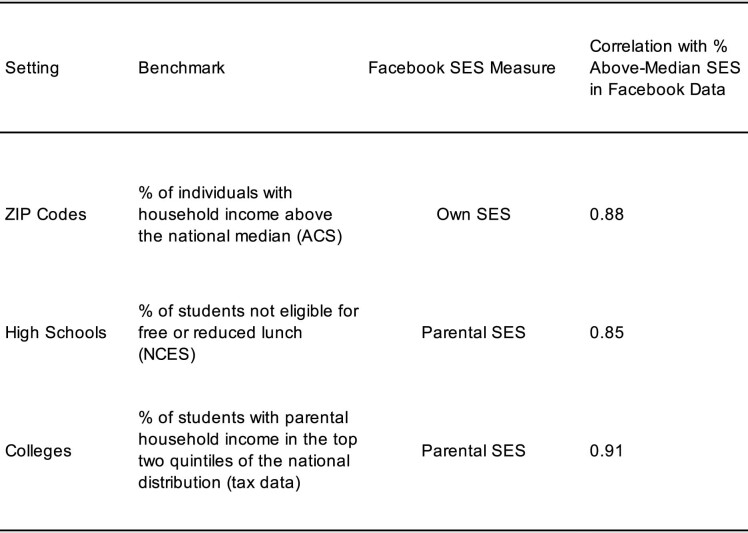
Validation of SES Predictions and Group Assignments using Publicly Available Data

This table evaluates the accuracy of the SES measures constructed in the Facebook data as well as the methods used to assign individuals to specific groups (i.e., specific neighbourhoods, high schools and colleges). Concretely, we compare the fraction of above-median-SES individuals in each ZIP code, high school and college to estimates of the fraction of high-income members of these groups from publicly available administrative data sources. In the first row, we correlate the fraction of households with above-median household income within each ZIP code, as calculated by ref. ^23^ using data from the 2014-2018 American Community Survey, with the estimated proportion of Facebook users in our primary sample with above-median SES. This correlation is weighted by ZIP code population based on the 2018 ACS. In the second row, for each public high school, we calculate the 5-year median of the fraction of students who are not eligible for free or reduced price lunch (based on the 2014-2018 NCES Common Core of Data). We correlate this measure with the fraction of students with above-median parental SES in the Facebook data, using individuals born between 2000 and 2004 to match the cohorts observed in the NCES data. This correlation is weighted by the number of students in grades 9 to 12, as reported in the NCES data. In the third row, we correlate the fraction of students with parental income in the top two quintiles of the national distribution in each college, as calculated by ref. ^46^ using tax records, with our corresponding estimates of the proportion of students with above-median parental SES from the Facebook data. This correlation is weighted by the number of students in the relevant college cohort. See Supplementary Information A for further details on the publicly available data sources.

**Extended Data Table 3:**
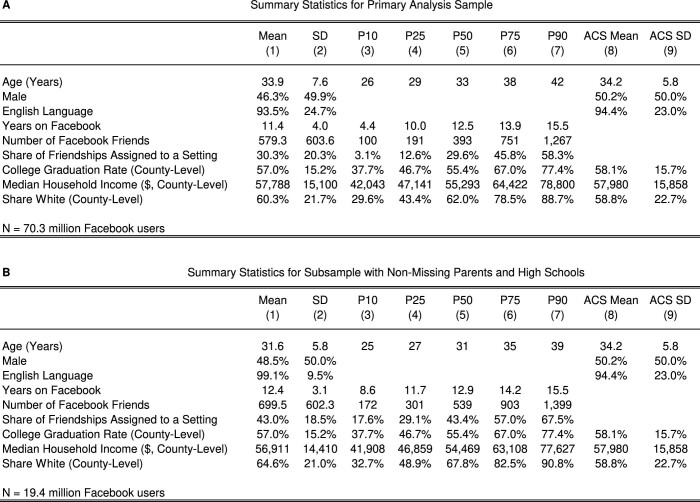
Summary Statistics for Analysis Samples

Panel A presents summary statistics for our primary analysis sample, which consists of individuals between the ages of 25 and 44 as of May 28, 2022 who reside in the United States, have been active on the Facebook platform at least once in the previous 30 days, have at least 100 U.S.-based Facebook friends, and for whom we are able to allocate at least one friend to a setting using the algorithm described in the ‘Variable Definitions’ section of Methods. Panel B replicates Panel A for the subsample used to measure childhood economic connectedness: individuals in the primary analysis sample whom we can link to parents with valid SES predictions (see Supplementary Information A.2 in our companion paper^7^) and who can be assigned to a high school (see Supplementary Information B.1). For each variable, columns 1-7 present means, standard deviations and selected percentiles. Columns 8 and 9 report means and standard deviations for the corresponding variables using the nationally representative 2012-2016 American Community Survey (ACS) for median household income and the 2014-2018 ACS for the remaining variables. Age and gender are self-reported by individuals. *English Language* refers to whether users set their language to English in the Facebook data and to the share of individuals who speak English only or speak English “very well” in the ACS data. *Years on Facebook* and *Number of Facebook Friends* are only observed in the Facebook data. In both panels, we report the number of Facebook friends within our primary analysis sample, as opposed to the total number of Facebook friends. *Share of Friends Assigned to a Setting *refers to the set of friends that we are able to assign to one of the six settings, as described in Supplementary Information B.2. The remaining variables are obtained from the 2014–2018 American Community Survey (ACS) and are assigned based on an individual’s residential county. The ACS means for these variables are population-weighted averages of the same county-level variables.

**Extended Data Table 4:**
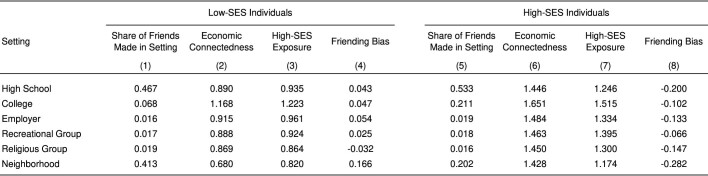
Friending Rates, Economic Connectedness, Exposure, and Bias by Setting and SES

This table shows the share of friends made in each of the six settings we consider along with mean levels of economic connectedness, high-SES exposure and friending bias in each setting. Columns 1–4 report these statistics for low-SES individuals and columns 5–8 report these statistics for high-SES individuals. See the notes to Fig. 2 and the description in Supplementary Information B.5 for definitions of each of these measures. In columns 1 and 5, we report average normalized friend shares—each individual’s raw friend share in a setting divided by the sum of their raw friend shares across the six settings—because the raw friend shares can sum to more than 1 due to the double counting of friendships assigned to multiple settings (Methods: ‘Variable Definitions’). Note that because exposure and bias covary across groups within settings, mean economic connectedness for each setting is not identical to the product of mean exposure and one minus mean friending bias at the setting level.

## Data Availability

The only data shared outside Meta were aggregate statistics on social capital (by county, ZIP code, etc.). We used methods from the differential privacy literature to add noise to these aggregate statistics to protect privacy while maintaining a high level of statistical reliability; see https://www.socialcapital.org for further details on these procedures.

## Code availability

Code supporting the findings of this study using the publicly released data is available at https://opportunityinsights.org.
