# Supplementary material for: Social capital II: determinants of economic connectedness
Source: Nature. 2022 Aug 1;608(7921):122–34. doi: 10.1038/s41586-022-04997-3 (PMC9352593; doi:10.1038/s41586-022-04997-3)
Supplement: Supplementary file 1 — Supplementary Information [file 41586_2022_4997_MOESM1_ESM.pdf]

nature portfolio

https://doi.org/10.1038/s41586-022-04997-3

Supplementary information

Social capital II: determinants of economic connectedness

In the format provided by the authors and unedited

Supplementary Information for "Social Capital II: Determinants of Economic Connectedness"

Raj Chetty*, Matthew O. Jackson*, Theresa Kuchler*, Johannes Stroebel*,
Nathaniel Hendren, Robert B. Fluegge, Sara Gong, Federico Gonzalez, Armelle Grondin,
Matthew Jacob, Drew Johnston, Martin Koenen, Eduardo Laguna-Muggenburg,
Florian Mudekereza, Tom Rutter, Nicolaj Thor, Wilbur Townsend, Ruby Zhang,
Mike Bailey, Pablo Barberá, Monica Bhole, and Nils Wernerfelt

Contents

A Supplementary Information on Data

A.1 Upward Mobility
A.2 High School Characteristics
A.3 College Characteristics

B Supplementary Methods

B.1 Assigning Facebook Users to Groups
B.2 Assigning Friendships to Settings
B.3 Adjusting for Underreporting
B.4 Treatment of Zero Friend Shares in Decompositions
B.5 Definitions of Statistics Reported in Tables and Figures
B.6 Regression Discontinuity Design: Specifications and Robustness

C Supplementary Discussion

C.1 Level of Aggregation and Exposure vs. Friending Bias
C.2 Relationship Between Exposure and Average Incomes Across Areas
C.3 Relationship Between Upward Mobility and Friending Bias
C.4 Friending Bias at Evanston Township High School: Ethnographic Evidence

* R.C., M.J., T.K., and J.S. contributed equally to this work.

A Supplementary Information on Data

In this section, we describe the external (non-Facebook) data we use in our analysis. Note that we do not link any external individual-level information to the Facebook data.

A.1 Upward Mobility

Data on economic mobility by Census tract and county are obtained from the publicly available Opportunity Atlas (Chetty et al. 2018). We define upward income mobility in each area as the average income percentile in adulthood of a child born to parents at the 25th percentile of the income distribution. We aggregate the Census tract data on upward mobility to the ZIP code (ZCTA) level using the number of children with below-median parental income as weights.

A.2 High School Characteristics

National Center for Education Statistics (NCES). Data on high schools and their characteristics are publicly available from the National Center for Education Statistics (NCES), the primary federal entity for collecting data from U.S. high schools. We obtain measures of the total number of students in grades 9 through 12, the percent of students eligible for free or reduced lunch (for public schools), and racial shares (Black, white, Asian, Hispanic, Native American) from the 2017-2018 Common Core of Data and Private School Universe Survey. We also use the surveys conducted in 1997-1998 and 2007-2008 to obtain lists of schools that have since closed, which we use to match individuals to schools used in our analyses (note, however, that we do not publicly release statistics on social capital for schools that have closed).

Civil Rights Data Collection (CRDC). We supplement the NCES data with data from 2015–2016 Civil Rights Data Collection (CRDC), a biennial survey of public schools on various civil rights indicators related to access to education. We obtain measures of the share of students in AP courses and the share of students enrolled in gifted or talented programs from the CRDC.

A.3 College Characteristics

Integrated Postsecondary Education Data System (IPEDS). Data on colleges and their characteristics, including racial shares (Black, white, Asian or Pacific Islander, Hispanic), are obtained from the 2013 Integrated Postsecondary Education Data System.

College Mobility Report Cards (Tax Data). We supplement the IPEDS data with data on the incomes of college students' parents, which are obtained from variables that were publicly released by Chetty et al. (2020). We also use the college tier variable from this dataset, which was constructed using IPEDS data.

B Supplementary Methods

This section provides details on six aspects of our methods: (1) identifying the groups in which individuals participate; (2) allocating friendships to these groups; (3) adjusting for under-reporting of group memberships; (4) the treatment of zero friend shares and zero exposure in decompositions, (5) formal definitions of various statistics shown in figures and tables; and (6) details on the regression discontinuity design used to identify the causal effects of changes in exposure on EC. Details on other aspects of our methods—such as estimation of socioeconomic status and computation of standard errors—are available in Supplementary Information B of Chetty et al. (2022).

B.1 Assigning Facebook Users to Groups

We begin by identifying the groups to which individuals in our analysis sample belong, focusing on the following six settings where friendships are formed: high schools, colleges, recreational groups, religious groups, workplaces, and neighborhoods (defined as ZIP codes). We describe how we assign individuals to specific groups in each of these settings in turn.

High schools. We assign individuals to high schools based on self-reported high schools, self-reported hometowns, and information from their social networks. We begin by matching self-reported high school names to the National Center for Education Statistics' (NCES) comprehensive surveys of U.S. public and private schools. We drop virtual schools, schools located in the five U.S. territories or on military bases abroad, and schools with fewer than 50 students. For individuals who self-report a common high school name (e.g., "Central High School") we only include that self-report if the individual also reports a hometown that matches the school's location. For the 3.3% of individuals with multiple self-reported high schools, we assign the school at which the individual has the greatest number of friends whose ages are within three years of the individual's own age. We exclude self-reported schools where individuals have fewer than 10 friends with ages within three years of their own (i,e., we do not include them when calculating EC, exposure, or bias for that school); however, we include these individuals in the potential set of friends for other individuals assigned to that school.

For people without a validated self-reported high school, we use their friendship network to impute their high school. For this imputation, we only consider friends who have a valid self-reported high school and who are within three years of the individual's age. We then calculate the ratio of an individual's friends in the high school where they have the most friends relative to the schools where they have the next most friends, and assign the user to the first high school if this ratio exceeds two (we further require that the individual has at least five friends in the first high school). We evaluate the accuracy of this imputation approach using the sample of users with validated self-reports. For users with a valid self-reported high school, the network-imputed high school matches the self-reported high school 97.4% of the time. Using this algorithm, we observe high schools for 74.9% of individuals in our analysis sample; 53.8% are assigned via self-reports and 21.1% via imputation based on their friendship network.

For the quasi-experimental analysis in the "Effects of Integration on Connectedness" section, we assign students to high school cohorts by collecting data on school entry cutoffs by year from Elder and Lubotsky (2009) and Bush and Zinth (2011) (in all other analyses, we define individuals' high school cohorts simply based on calendar years of birth for simplicity). See Supplementary Table 2 for a list of the entry date cutoffs we use in our analysis. The average high school cohort has 115 users assigned to it (Supplementary Table 3). To ensure that our quasi-experimental estimates are not biased by imputing high schools based on friendship networks, we verify that the results in Extended Data Figure 4 remain similar when restricting to individuals for whom we observe high schools based solely on self-reports.

Colleges. To assign people to colleges, we begin by matching individuals' self-reported colleges to the IPEDS directory. We drop online colleges as well as those that do not appear in the Carnegie Classification. For the 17.8% of individuals with multiple self-reported colleges conditional on being assigned a college, we use the one with the maximum number of friends (restricting attention friends within three years of the individual's age who have a valid self-reported college). We exclude self-reported colleges where individuals have fewer than 10 friends with ages within three years of their own (i,e., we do not include them when calculating EC, exposure, or bias for that college); however, we include these individuals in the potential set of friends for other individuals assigned to that college. We observe a college for 42.9% of individuals in our analysis sample, and the average college has 530 users per cohort.

Recreational groups. To analyze friendships formed in recreational groups, we use algorithms that classify Facebook groups by topic based on their titles. Since our goal is to capture recreational

activities that can facilitate real-life friendships, we restrict attention to groups classified as sports, fitness, performing arts, crafts, and literature. We also exclude any groups related to the buying and selling of items. Among these groups, we impose two further restrictions to increase the likelihood that members of the group have met in person (i.e., not just virtually). First, we only consider groups with between 10 and 3,000 members in our primary analysis sample. Second, we require that at least 80% of group members must reside in a single commuting zone. We exclude members who do not live in this modal commuting zone. For the 45.9% of users belonging to multiple recreational groups that satisfy all of these restrictions, we select the recreational group with the maximum number of friends.[1] We assign 29.8% of individuals in our analysis sample a recreational group using this approach, each of which has 17 users on average assigned to it.

Faith-based (Religious) Groups. We use regular expressions to identify Facebook pages that are faith-based (excluding pages containing education, conference, event, media, or music-related phrases), and restrict attention to pages with an admin-reported U.S. address. We also identify faith-based Facebook groups, which are classified based on the title of the group and other group characteristics. When identifying faith-based pages or groups, we only consider pages with between 20 and 2,000 likes and groups with between 20 and 2,000 members.

We assign individuals to pages based on page likes and to groups based on the individuals joining the particular group. We only assign individuals to pages or groups that are located in the individual's own commuting zone. In the case of assignment to multiple pages or groups, we prioritize those located in the individual's county, and within that subset select the page or group with the highest number of friends. We identify a faith-based page or group for 17.9% of users using this approach. On average, each faith-based page or group has 39 individuals in our analysis sample.

For convenience, we refer to faith-based groups identified using this approach as "religious groups" throughout the paper. Note that our classification does not infer an individual's religion: it is focused on whether friendships were formed in a faith-based group, rather than whether an individual belonged to or identified with a particular religion.

Workplaces. We use self-reported data on individuals' Facebook profiles to assign them to workplaces. We apply three restrictions to increase data quality. First, we remove self-reported employers that are clearly fictional or contrived (e.g., "The Krusty Krab", "None of your business", and "Businessperson"). Next, we remove employment that does not involve external interactions such as self-employment, stay-at-home parenting, and blogging. Finally, we require the linked employer page to contain at least 10 other employees between the ages of 22 and 65 from the same county as the user (but not the same county as the employer page, since regional branches may not be listed separately). For the 9.5% of users with multiple valid employers (among those with at least one valid employer), we select the employer with the highest number of friends.

We observe an employer for 20.9% of individuals in our analysis sample. Each employer has on average 45 users assigned to them. As expected given the skewed distribution of the size of firms, some employers are matched to far more people than others; the distribution exhibits substantial dispersion, with a standard deviation of 881.

Neighborhoods. We use ZIP code tabulation areas (ZIP codes) to represent neighborhoods. Because Facebook users only have one current ZIP code, they are mechanically matched to a single neighborhood. If an individual has fewer than 10 friends in their assigned neighborhood, we define their neighborhood as missing (i,e., we do not include them when calculating EC, exposure, or bias for that neighborhood); however, we include these individuals in the potential set of friends for other individuals assigned to that neighborhood.

[1] As a robustness check to the method of selecting only the top group by friendship links, we also calculate metrics of friendship behavior using two alternative approaches: taking the union of all groups and random assignment of groups. For recreational groups, the individual-level EC correlations between just taking the top group vs. the union of all groups and random assignment of groups are 0.98 and 0.96. The corresponding individual-level group bias correlations are 0.88 and 0.85. We perform the same exercise for other settings and find similarly high correlations.

Note that when we measure economic connectedness for neighborhoods in this paper, we refer to the share of high-SES friends among friends who live *within* an individual's own ZIP code. This differs from the neighborhood-level economic connectedness measures analyzed in our first paper (Chetty et al. 2022), which are based on *all* the friends of people living in a given neighborhood, irrespective of whether those friends live in the same neighborhood.

Supplementary Table 3 shows statistics on the share of individuals we are able to assign to a group in each of these settings, both overall and by SES. Across all settings (except neighborhoods), we assign high-SES individuals to groups at a higher rate than low-SES groups. These differences are largely due to differences in participation rates by SES; for instance, high-SES people are more likely to have attended college and be working, and hence it is natural that they are more likely to be assigned to colleges or employers. Consistent with this view, our statistics on the share of high-SES individuals by group are highly correlated with external, publicly available data (Extended Data Table 2). Nevertheless, some of the differences in rates of group membership by SES may be driven by differences in reporting rates. To assess whether such reporting differences might bias our results, we develop an algorithm to adjust for differential under-reporting by SES using external data on group memberships. we describe this algorithm in Section B.3 below, and show that our results remain similar with this correction.

B.2 Assigning Friendships to Settings

After assigning individuals to groups, we allocate their friendships to these groups. A friendship is assigned to a particular group (e.g., a high school or a recreational group) when both individuals in the friendship are members of the group.

When friends appear in multiple settings—for example, when two friends share a common recreational group and a common employer—one could either attempt to assign the friendship to the setting that sparked the friendship or assign the friendship to all settings in which it appears. We adopt the latter agnostic approach. For example, if a user and their friend share both a high school and a recreational group, then that friendship link is counted in both settings. While this approach overweights friendships that appear in multiple settings, it avoids the need to adjudicate between settings and may appropriately place more weight on more important friends with whom one has contact in multiple settings. Using this procedure, we assign 30% of friendships to at least one of the six settings.

In Supplementary Table 3b, we report the percentage of friends from each setting that overlaps with another setting. For instance, 27.6% of college friends are also high school friends, and 36.1% of recreational group friends are neighborhood friends (i.e., they live in the same ZIP code).

B.3 Adjusting for Underreporting

The procedure described above to assign friendships to groups likely underestimates the raw number of friends made in each setting because we rely on user self-reports and conservative imputation procedures. We evaluate the sensitivity of our results to such under-reporting by implementing adjustments for the number of friends made in each setting in two steps.

First, we estimate reporting rates for each setting by dividing the share of individuals assigned to a setting in the Facebook data by the "true" share of individuals in the population who participate in that setting based on external data sources (e.g., the share of people who are members of religious groups). For high school attendance, college attendance, and employment rates, we use data from the 2014–2018 American Community Survey 5-year estimates (U.S. Census Bureau 2017) at the state $\times$ SES level. We use graduation rather than attendance rates to benchmark high school and college participation because Facebook users might be more likely to report their high school/college if they graduated from it. For religious group participation, we use data from the Pew Research Center (Pew Research Center 2014) at the state level. For neighborhoods, there is no under-reporting because we observe ZIP codes for everyone. We are unable to obtain reliable

estimates of true participation rates for recreational groups and therefore do not make adjustments for those groups. We measure participation rates at the finest available geographic/SES level for each setting. For example, we calculate reporting rates at the state × SES (above/below median) level for colleges, but we are only able to calculate a state-level reporting rate for religious groups as we do not have data on religious group attendance by SES and state.

Second, we use these reporting rates to correct the average share of friends at the setting × state × SES level. For each state t and SES type e, we define an adjusted share of friends in each setting s as:

$$\text{Adjusted Friend Share}_{t,e,s} \equiv \frac{\text{Raw Friend Share}_{t,e,s}}{r_{t,e,s} E[r_{t,e(j),s} | j \in N_{t,e,s}]}, \tag{1}$$

where $r_{t,e,s}$ is the reporting rate estimated for state t, SES type e, and setting s and $E[r_{t,e(j),s}|j \in N_{t,e,s}]$ is the average reporting rate in that setting for the set of friends $N_{t,e,s}$ made in that setting by people of SES type e (noting that within a setting, high and low-SES friends may have differing reporting rates). Intuitively, we inflate raw friend shares by the product of these two reporting rates to account for the fact that a friendship link is missed if either the individual or the friend does not report their group membership. Finally, we normalize the adjusted shares of friends across the six settings to sum to 1 within each state × SES type.

In Supplementary Figure 4, we use these adjusted friend shares instead of the raw friend shares as weights in the decompositions of differences in economic connectedness. The results are similar to those obtained with the raw shares used in our baseline analysis, suggesting that underreporting of friendships does not significantly bias our estimates.

B.4 Treatment of Zero Friend Shares in Decompositions

In this section, we describe how we define the share of high-SES friends $f_{H,i,g}$ in groups where an individual i makes no friends for the decompositions discussed in the Decomposing Economic Connectedness section of Methods. Formally, $f_{H,i,g}$ is undefined in groups that i does not belong to. However, since $\phi_{i,g} = 0$ for any such group, our calculations are unaffected by how $f_{H,i,g}$ is defined for those groups; we therefore set $f_{H,i,g} = 0$ for groups g of which i is not a member.

We also exclude groups having $w_{H,g} = 0$ from G because friending bias $(1 - \frac{f_{H,i,g}}{w_{H,g}})$ is not well-defined for such groups. Only 0.01% of group memberships have $w_{H,g} = 0$ when "high-SES" is defined as having above-median SES; thus, our results would be very similar if we were to use a different convention in such cases.

B.5 Definitions of Statistics Reported in Tables and Figures

In this section, we provide formal definitions of the statistics reported in Figures 1–6, which report and decompose measures of economic connectedness, exposure, and friending bias at different levels of aggregation. Let i index individuals in our sample and g index groups. Let $G(i)$ denote the set of groups of which i is a member and in which i makes at least one friend.

Figure 1: Figure 1 plots friending shares by setting for individuals with different SES. The settings are $s \in${college, employer, high school, neighborhood, recreational group, religious group}. Individuals can only be part of one group in a given setting (e.g., individuals can only be assigned one high school) and the share of friends made in group g is $\phi_{i,g} = 0$ for all groups g of which i is not a member. We compute average friending rates $\phi_{v,s}$ in each setting s separately for individuals in different SES ventiles $v \in \{1, 2, \ldots, 20\}$. Let $v(i)$ denote i's SES ventile and $s(g)$ denote the setting of a group g. Then, setting-level friending shares by SES ventile are:

$$\phi_{v,s} = \frac{1}{N_v} \sum_{v(i)=v} \sum_{s(g)=s} \phi_{i,g}, \tag{2}$$

where N_v denotes the number of individuals who are in SES ventile v. Figure 1 plots these friending shares $\phi_{v,s}$ normalized by the overall mean friend shares in each setting. That is, for each SES ventile v and setting s, we plot $\phi_{v,s}/(\sum_{v=1}^{20} \phi_{v,s}/20)$.

Figure 2: Figure 2 plots mean EC, exposure, and friending bias by setting, separately for individuals with below-median-SES ($SES = L$) and above-median-SES ($SES = H$). In what follows, note that we define the share of high-SES friends $f_{H,i,g} = 0$ for all groups g of which i is not a member (see Supplementary Information B.4 above). Let $N_{SES,s}$ denote the number of individuals who have a certain SES and make at least one friend in setting s, e.g., $N_{L,college}$ refers to the number of low-SES individuals in our analysis sample who make at least one friend in college.

Panel A: We compute average economic connectedness to high-SES individuals, $\text{EC}_{H,SES,s}$, separately for $SES \in \{L, H\}$ types in each setting s. These values are computed by averaging individuals' shares of high-SES friends, corresponding to $f_{H,i,g}$ from equation (2) in Methods, in the groups that belong to setting s, i.e. $s(g) = s$, over all individuals of type SES who are assigned to a group in setting s. This expression is then normalized by the share of high-SES individuals in the national population, $w_H = 0.5$:

$$\text{EC}_{H,SES,s} = \frac{1}{N_{SES,s} w_H} \sum_{i \in SES} \sum_{s(g)=s} f_{H,i,g}. \tag{3}$$

The bar chart plots $\text{EC}_{H,L,s}$ and $\text{EC}_{H,H,s}$ separately for each setting.

Panel B: We compute average exposure to high-SES individuals, $\text{Exposure}_{H,SES,s}$, separately for $SES \in \{L, H\}$ types in each setting s. Note that because we require individuals to attend high school or college within three cohorts of each other in order to be deemed high school or college friends, we measure individuals' exposure to high-SES peers in their high schools and colleges to be the share of high-SES individuals in the cohorts that lie within three years of the individual's own birth cohort (defining cohorts based on calendar years). In these cases, exposure therefore depends on the particular cohort that an individual is in. For example, the exposure in college of a student in cohort c, which we denote by $\text{Exposure}^{c}_{H,\text{college}}$, is twice the share of high-SES students in cohorts $\{c-3, c-2, \ldots, c+3\}$. Let $c(i)$ denote individual i's cohort and $w^{c(i)}_{H,g}$ denote the share of high-SES individuals in the relevant set of three cohorts around c.[2] In the other settings (neighborhoods, workplaces, recreational and religious groups), we do not impose cohort restrictions to assign friendships, and hence every group member has the same exposure and the cohort superscript can be ignored.

We define $\text{Exposure}_{H,SES,s}$ as the mean share of high-SES peers in the group g to which individual i belongs in setting ($s(g) = s$), averaging over all individuals of type SES who have at least one friend in setting s. We then normalize this expression by the share of high-SES individuals in the national population, $w_H = 0.5$:

$$\text{Exposure}_{H,SES,s} = \frac{1}{N_{SES,s} w_H} \sum_{i \in SES} \sum_{\substack{g \in G(i) \\ s(g)=s}} w^{c(i)}_{H,g}. \tag{4}$$

The bar chart plots $\text{Exposure}_{H,L,s}$ and $\text{Exposure}_{H,H,s}$ separately for each setting.

Panel C: We compute average friending bias with respect to high-SES individuals, $\text{Friending Bias}_{H,SES,s}$, separately for $SES \in \{L, H\}$ types in each setting s. At the individual level, friending bias is defined as the ratio of an individual's share of high-SES friends to their share of high-SES peers $\frac{f_{H,i,g}}{w^{c(i)}_{H,g}}$

[2]We also measure economic connectedness and friending bias only based on friends in an individual's cohort and the three surrounding cohorts. However, since the high-SES friend share $f_{H,i,g}$ is already defined at the individual level rather than the group level, we can simplify notation and do not include an additional superscript c for those expressions.

in an individual's group that belongs to setting s ($s(g) = s$). We then compute mean friending bias by SES type and setting by averaging individual friending bias over all individuals of type SES who have at least one friend in setting s:

$$\text{Friending Bias}_{H,SES,s} = 1 - \frac{1}{N_{SES,s}} \sum_{i \in SES} \sum_{\substack{g \in G(i) \\ s(g)=s}} \frac{f_{H,i,g}}{w_{H,g}^{c(i)}}. \tag{5}$$

The bar chart plots $\text{Friending Bias}_{H,L,s}$ and $\text{Friending Bias}_{H,H,s}$ separately for each setting.

Panel D: As in Panel C, we first compute average friending bias with respect to high-SES individuals, $\text{Friending Bias}_{H,L,s}$, for low-SES individuals in each setting s, restricting the sample to members of a religious group. Let R be the set of individuals i who are members of religious groups (i.e., any i such that $\exists g \in G(i)$ with $s(g) =$ religious group). Let $N_{L,s,R}$ denote the number of low-SES religious group members who are also part of a setting s and make at least one friend in setting s. The values in the bars are given by computing mean friending bias in each setting as in Panel C using the subset of low-SES individuals who are part of a religious group ($i \in L \cap R$), and then subtracting the mean friending bias in the religious group setting for those individuals. Formally, we plot the following for all non-religious group settings s, using "rel" as an abbreviation for "religious group":

$$\begin{aligned} &\text{Friending Bias}_{H,L,s,R} - \text{Friending Bias}_{H,L,\text{rel}} \\ &= \left(1 - \frac{1}{N_{L,s,R}} \sum_{i \in L \cap R} \sum_{\substack{g \in G(i) \\ s(g)=s}} \frac{f_{H,i,g}}{w_{H,g}^{c(i)}}\right) - \left(1 - \frac{1}{N_{L,\text{rel}}} \sum_{i \in L \cap R} \sum_{\substack{g \in G(i) \\ s(g)=\text{rel}}} \frac{f_{H,i,g}}{w_{H,g}}\right) \\ &= \frac{1}{N_{L,\text{rel}}} \sum_{i \in L \cap R} \sum_{\substack{g \in G(i) \\ s(g)=\text{rel}}} \frac{f_{H,i,g}}{w_{H,g}} - \frac{1}{N_{L,s,R}} \sum_{i \in L \cap R} \sum_{\substack{g \in G(i) \\ s(g)=s}} \frac{f_{H,i,g}}{w_{H,g}^{c(i)}} \end{aligned} \tag{6}$$

Figure 3A: To construct Figure 3A, we begin by defining a representative-agent notion of economic connectedness based on the product of the setting-specific average friending rates, average exposure, and average bias for people in that SES group:

$$EC_{H,SES} \equiv \sum_s \phi_{SES,s} \times \text{Exposure}_{H,SES,s} \times (1 - \text{Friending Bias}_{H,SES,s}), \tag{7}$$

where the variables $\text{Exposure}_{H,SES,s}$ and $\text{Friending Bias}_{H,SES,s}$ are defined as in equations (4) and (5) above, and $\phi_{SES,s} = (1/N_{SES}) \sum_{i \in SES} \sum_{s(g)=s} \phi_{i,g}$. This equation is a representative-agent analog of equation (2) in Methods, in which $EC_{H,SES}$ is the product of setting-level means of friending shares, exposure, and bias, which can differ from an average of individual-level EC. $EC_{H,SES}$ may also differ from the across-setting mean of $EC_{H,SES,s}$ as defined above. The reason is that multiplying setting-level averages ignores any covariance between friend shares, exposure, and friending bias at the individual level.[3]

We use equation (7) to decompose why $EC_{L,SES}$ differs from $EC_{H,SES}$.

Bar 1: This bar shows $EC_{H,L}$ as defined in equation (7).

[3] As a reference, the top bar in Figure 3A shows that the EC of low-SES individuals is 0.83 on average, while the bottom bar shows that the corresponding value is 1.53 for high-SES individuals—corresponding to a gap in EC of 0.7. In addition to the differences driven by the covariance terms just described, both numbers also differ slightly from the EC gap between low- and high-SES individuals of 1.4 − 0.78 = 0.63 reported in Chetty et al. (2022), since that number is based on all friends, while the numbers in this paper are only based on friendships that we can allocate to a group where they were likely formed.

Bar 2: In this bar, we set the friending shares of low-SES users to be equal to those of high-SES individuals, i.e., $\forall s$, set $\phi_{L,s} = \phi_{H,s}$. As such, this bar plots $\sum_s \phi_{H,s} \times \text{Exposure}_{H,L,s} \times (1 - \text{Friending Bias}_{H,L,s})$.

Bar 3: In this bar, we keep the equated friending shares and also equate the setting-level exposure of low-SES individuals to that of high-SES individuals, i.e., $\forall s$, set $\text{Exposure}_{H,L,s} = \text{Exposure}_{H,H,s}$. This bar plots $\sum_s \phi_{H,s} \times \text{Exposure}_{H,H,s} \times (1 - \text{Friending Bias}_{H,L,s})$.

Bar 4: In this bar, instead of equating exposure, we equate bias, i.e., $\forall s$, set $\text{Friending Bias}_{H,L,s} = \text{Friending Bias}_{H,H,s}$, and plot $\sum_s \phi_{H,s} \times \text{Exposure}_{H,L,s} \times (1 - \text{Friending Bias}_{H,H,s})$.

Bar 5: Lastly, in the bottom bar, we report $EC_{H,H}$ as defined in equation (7), which is equivalent to equating friending shares, exposure, and bias simultaneously, i.e., $\sum_s \phi_{H,s} \times \text{Exposure}_{H,H,s} \times (1 - \text{Friending Bias}_{H,H,s}) = EC_{H,H}$.

Figure 3B: In Figure 3B, we conduct a similar counterfactual exercise across ZIP codes. We decompose the economic connectedness in ZIP codes with the highest vs. the lowest 20% of average economic connectedness among low-SES individuals to understand what drives differences in EC among low-SES individuals across ZIP codes. To this end, we define representative-agent measures of mean friending shares, exposure, and bias for low-SES individuals in different ZIP-code-EC-quintiles and settings. Let $z(i)$ denote i's ZIP code.
We divide ZIP codes z into quintiles $q \in \{1, 2, \ldots, 5\}$ in terms of their ZIP-code-level economic connectedness, $EC_{H,L,z} = \frac{1}{N_{L,z} w_H} \sum_{\substack{i \in L \\ z(i)=z}} \sum_{g \in G(i)} \phi_{i,g} \times f_{H,i,g}$, weighted by the number of low-SES individuals in each ZIP code. Let $q(z)$ denote the EC quintile of ZIP code z. Let $N_{L,q}$ denote the number of low-SES individuals living in ZIP codes that are in EC quintile q and $N_{L,s,q}$ the number of low-SES individuals in those ZIP codes who additionally make at least one friend in setting s. We then define the following setting $\times$ quintile measures of friend shares, exposure, and friending bias among low-SES individuals:

$$\phi_{L,s,q} = \frac{1}{N_{L,q}} \sum_{\substack{i \in L \\ (q \circ z)(i) = q}} \sum_{s(g)=s} \phi_{i,g} \tag{8}$$

$$\text{Exposure}_{H,L,s,q} = \frac{1}{N_{L,s,q} w_H} \sum_{\substack{i \in L \\ (q \circ z)(i) = q}} \sum_{\substack{g \in G(i) \\ s(g)=s}} w_{H,g}^{c(i)}, \tag{9}$$

$$\text{Friending Bias}_{H,L,s,q} = 1 - \frac{1}{N_{L,s,q}} \sum_{\substack{i \in L \\ (q \circ z)(i) = q}} \sum_{\substack{g \in G(i) \\ s(g)=s}} \frac{f_{H,i,g}}{w_{H,g}^{c(i)}}. \tag{10}$$

We then decompose the following equation:

$$EC_{H,L,q} \equiv \sum_s \phi_{L,s,q} \times \text{Exposure}_{H,L,s,q} \times (1 - \text{Friending Bias}_{H,L,s,q}). \tag{11}$$

Similar to the previous decomposition, note that the representative-agent measure $EC_{H,L,q}$ here may differ from the average of individual-level $IEC_{H,i}$ among low-SES individuals across ZIP codes in the bottom quintile of average economic connectedness, because of individual-level covariances between friend shares, exposure, and friending bias.
We use equation (11) to decompose why $EC_{H,L,1}$ differs from $EC_{H,L,5}$.

Bar 1: This bar shows $EC_{H,L,1}$ as defined in equation (11).

Bar 2: In this bar, we set the friending shares of low-SES individuals in low-EC ZIP codes to be those of low-SES individuals in high-EC ZIP codes, i.e., $\forall s$, set $\phi_{L,s,1} = \phi_{L,s,5}$. As such, this bar shows $\sum_s \phi_{L,s,5} \times \text{Exposure}_{H,L,s,1} \times (1 - \text{Friending Bias}_{H,L,s,1})$.

Bar 3: In this bar, we keep the equated friending shares and also equate the setting-level exposures of low-SES individuals in low-EC ZIP codes to those of low-SES individuals in high-EC ZIP codes, i.e., $\forall s$, set $\text{Exposure}_{H,L,s,1} = \text{Exposure}_{H,L,s,5}$. This bar plots $\sum_s \phi_{L,s,5} \times \text{Exposure}_{H,L,s,5} \times (1 - \text{Friending Bias}_{H,L,s,1})$.

Bar 4: In this bar, instead of equating exposure, we equate bias, i.e., $\forall s$, set $\text{Friending Bias}_{H,L,s,1} = \text{Friending Bias}_{H,L,s,5}$, and plot $\sum_s \phi_{L,s,5} \times \text{Exposure}_{H,L,s,1} \times (1 - \text{Friending Bias}_{H,L,s,5})$.

Bar 5: In this bar, we report $EC_{H,L,5}$. This is equivalent to equating friending shares, exposure, and bias simultaneously, i.e., $\sum_s \phi_{L,s,5} \times \text{Exposure}_{H,L,s,5} \times (1 - \text{Friending Bias}_{H,L,s,5}) = EC_{H,L,5}$.

Figure 4: Figure 4 presents county-level and ZIP-level maps of exposure and bias for low-SES individuals. We define the following measures, calculated over all individuals whom we can assign to a setting. The differences relative to the measures in Figure 2 are that here we focus on $SES = L$, aggregate over the setting dimension s, and add a geographic dimension $a \in \{\text{county, ZIP}\}$. Let $N_{L,a}$ denote the number of low-SES individuals in area a (county or ZIP) and $a(i)$ denote i's area.

Panels A-B: These panels plot area-level (county- or ZIP-code-level) means of exposure based on the groups to which low-SES individuals belong. These area-level measures of the exposure of low-SES to high-SES individuals are computed by first calculating individuals' exposure as the sum of the product of their friend shares in each group $\phi_{i,g}$ and exposure in the individual's cohort-group, $\text{Exposure}^{c(i)}_{H,g}$, and then averaging over all low-SES individuals in an area a ($i \in L : a\,(i) = a$):

$$\text{Exposure}_{H,L,a} = \frac{1}{N_{L,a}} \sum_{\substack{i \in L \\ a(i)=a}} \sum_{g \in G(i)} \phi_{i,g} \times \text{Exposure}^{c(i)}_{H,g}. \tag{12}$$

Panels C–D: These panels plot area-level (county- or ZIP-code-level) means of friending bias based on the groups to which low-SES individuals belong and the friendships made in those groups. These area-level measures of friending bias are computed by first calculating individual-level friending bias as the sum of the product of their friend shares in each group $\phi_{i,g}$ and friending bias in that group, $\text{Friending Bias}_{H,i,g}$, and then averaging over all low-SES individuals in an area a ($i \in L : a\,(i) = a$):

$$\text{Friending Bias}_{H,L,a} = \sum_{\substack{i \in L \\ a(i)=a}} \sum_{g \in G(i)} \phi_{i,g} \times \text{Friending Bias}_{H,i,g}. \tag{13}$$

Figure 5: In Figure 5, we report exposure and bias estimates for colleges and high schools. These measures are obtained by averaging low-SES individuals' exposure and bias over all low-SES individuals in each group who make at least one friend in that group. Specifically, for a particular high school or college u[4]:

$$\text{Exposure}_{H,L,u} = \frac{1}{N_{L,u}} \sum_{i \in L} \sum_{u \in G(i)} \text{Exposure}^{c(i)}_{H,u} \tag{14}$$

and

$$\text{Friending Bias}_{H,L,u} = \frac{1}{N_{L,u}} \sum_{i \in L} \sum_{u \in G(i)} \text{Friending Bias}_{H,i,u}. \tag{15}$$

[4] These definitions of exposure and friending bias apply more generally to groups u in all settings $s \in$ {college, employer, high school, neighborhood, recreational group, religious group}. For example, Extended Data Table 1a presents a variance decomposition using these group-level measures and Extended Data Table 4 reports summary statistics on exposure, friending bias, and economic connectedness for all settings. Supplementary Figure 1 analyzes friending bias across groups within different settings.

We can also compute the school- or college-level average economic connectedness to high-SES students among low-SES students. This measure is given as individuals' economic connectedness at that school, averaged over all low-SES individuals who attend the school and make at least one friend in that school:

$$\mathrm{EC}_{H,L,u} = \frac{1}{N_{L,u} w_H} \sum_{i \in L} \sum_{u \in G(i)} f_{H,i,u} \tag{16}$$

Figure 6: In Figure 6, we analyze the causal effects of being assigned to a high-school cohort with more high-SES peers on economic connectedness for low-SES students. We focus on connections between children with parents in the lowest and highest SES quintiles (rather than below vs. above median SES). Hence, we define different SES types $SES \in \{L', H'\}$, with L' denoting the set of bottom-quintile-SES individuals and H' denoting the set of top-quintile-SES individuals.

We first measure rates of exposure to high-SES students by school cohort. To do so, we first assign individuals to school cohorts based on their birth dates relative to the school entry cutoff date for their school (see Supplementary Table 2). We then define exposure in a given cohort c of high school u as $\mathrm{Exposure}^c_{H',u} = \frac{w^c_{H',u}}{w_{H'}}$, where $w^c_{H',u}$ is the share of top-quintile-SES people in cohort c, and $w_{H'} = 0.2$. Because these figures exploit cohort-level fluctuations in peers, we define exposure differently from how it is defined in Figure 2b: here, $w^c_{H',u}$ refers to exposure only *within* cohort c itself, rather than in cohorts $\{c-3, c-2, \ldots, c+3\}$.

We define the cohort-level deviation of exposure as mean exposure for the relevant cohort c in a given school u minus the mean for all other cohorts in the same school, weighting by the number of bottom-quintile-SES students in each cohort:

$$\Delta\mathrm{Exposure}^c_{H',u} = \mathrm{Exposure}^c_{H',u} - \sum_{j \neq c} p_{u,j} \times \mathrm{Exposure}^j_{H',u} \tag{17}$$

where $p_{u,j}$ is the share of bottom-quintile-SES students in cohort j out of all bottom-quintile-SES students in school u not in cohort c.

In Panel A, we examine the relationship between $\Delta\mathrm{Exposure}^c_{H',u}$ and an analogous cohort-level deviation measure for economic connectedness. To construct the latter, we start by defining $f_{H',i,u}$ as the fraction of top-quintile-SES friends that individual i makes in school u and cohort $c(i)$. Let $N_{L',u,c}$ denote the number of bottom-quintile-SES students in school u and cohort c. Then, the cohort-level deviation of economic connectedness is

$$\Delta\mathrm{EC}^c_{H',L',u} = \frac{1}{N_{L',u,c} w_{H'}} \sum_{\substack{i \in L' \\ c(i)=c}} f_{H',i,u} - \sum_{j \neq c} p_{u,j} \left(\frac{1}{N_{L',u,j} w_{H'}} \sum_{\substack{i \in L' \\ c(i)=j}} f_{H',i,u} \right). \tag{18}$$

Panel A presents a binned scatterplot of $\Delta\mathrm{EC}^c_{H',L',u}$ vs. $\Delta\mathrm{Exposure}^c_{H',u}$ and corresponding slope estimate from an OLS regression.

In Panel B, we construct a measure of friending bias using data from all cohorts excluding the focal cohort c as follows:

$$\text{Friending Bias}^{-c}_{H',L',u} = 1 - \frac{1}{N_{L',u,-c}} \sum_{\substack{i \in L' \\ c(i) \neq c}} \frac{f_{H',i,u}}{w^{c(i)}_{H',u}} \tag{19}$$

where $N_{L',u,-c}$ is the number of bottom-quintile-SES students in school u not in cohort c. We use this leave-out measure of bias to divide school-cohort cells into ten deciles. We then estimate OLS

regressions analogous to that in Panel A using the cohort-school cells in each of the ten deciles separately. Finally, we plot the slopes from these ten regressions vs. the mean level of (leave-out) friending bias in each decile.

B.6 Regression Discontinuity Design: Specifications and Robustness

In the main text, we use a regression discontinuity design to estimate the causal effect of exposure to high-SES peers on economic connectedness in high schools. Here, we discuss the estimating equations and identification assumptions underlying this design.

We begin from data at the individual level and define economic connectedness EC_i for individual i as five times the share of their high school friends in their cohort who have parental SES in the top quintile of the national parental SES distribution. We then consider every pair of adjacent cohorts, letting cohort 1 be the cohort with greater high-SES exposure and cohort 2 the other cohort. For each student in cohort 1, let d_i denote the number of days between their birthdate and the cutoff date between the two cohorts (using the cutoffs defined in Supplementary Table 2). For students in cohort 2, define the number of days between their birthdate and the cutoff between the two cohorts analogously, but code it to be negative. Each student in the two cohorts is then entered into the dataset with an accompanying d_i, positive for students in cohort 1 and negative for those in cohort 2.

Since we do this for every pair of cohorts, students will appear twice in the dataset (except for those in the first and last cohorts), once with d_i that is defined relative to the cutoff date defining the cohort younger than the student and once with d_i defined relative to the cutoff defining the cohort older than the student. Depending on the comparison of the student's cohort's own high-SES share relative to the preceding and following cohorts, these values of d_i could both be positive, negative, or be of different signs. For example, consider a student i in cohort $c(i)$. If i is born 15 days after the cutoff from cohort $c-1$ and the share of high-SES students in $i's$ school is lower in cohort c than $c-1$, then $d_i = -15$ for $(c-1, c)$ pair of cohorts. If the share of high-SES students is greater in cohort c than $c+1$, then $d_i = +350$ for the $(c, c+1)$ cohort pair.

We collapse the resulting individual-level dataset into cell means, defining cells by three dimensions: (1) quartile of changes in exposure across cohorts ($\triangle e_q$), (2) quartile of friending bias estimated using data from other cohorts excluding the two focal cohorts (b_q), and (3) days between individuals' dates of birth and school entry cutoff dates, defined as above (d).

Using data for a given set of exposure change by bias $\{\triangle e_q, b_q\}$ cells, we estimate regression specifications of the following form:

$$EC_{\triangle e_q, b_q, d} = \beta_0 + \beta_1 T_d + \beta_2 d + \beta_3 T_d \times d + \epsilon_{e_q, b_q, d}, \tag{20}$$

weighting by the number of individuals in each $\triangle e_q \times b_q \times d$ cell.

In equation (20), the outcome variable $EC_{\triangle e_q, b_q, d}$ is average EC in each $\triangle e_q \times b_q \times d$ cell. The indicator variable $T_d = 1\{d > 0\}$ takes a value of 1 when individuals fall on the side of the entry cutoff that has greater high-SES exposure. The coefficient of interest is β_1, which is an estimate of how much the economic connectedness of low-SES individuals changes in response to the jump in high-SES exposure. In Extended Data Figure 4a, we focus on pairs of adjacent cohorts where the magnitude of the jump in the share of high-parental-SES students lies in top quartile of the distribution of changes in high-SES shares ($\triangle e_q = 4$); in Extended Data Figure 4b, we report estimates for all four quartiles of the change in high-SES shares across cohorts. See the notes to Extended Data Figure 4 for additional details on implementation of this specification.

The key assumption for (20) to yield an unbiased estimate of β_1 is that only high-SES exposure changes at the cutoff and that individuals on either side of the cutoff are similar on all other relevant characteristics ϵ that may affect EC. To evaluate this identification assumption, we assess whether observable characteristics are balanced on the two sides of the cutoff. In Supplementary Figure 7,

we replicate Extended Data Figure 4a replacing EC_i with individual i's total number of friends and sex as the outcome variables. Both the number of friends and share female trend smoothly around the cutoff, supporting the validity of the identification assumption.

In the baseline regression specification used in Extended Data Figure 4a, we include students within 200 days of the entry cutoff ($-200 \leq d_i \leq 200$). To evaluate the sensitivity of our estimates, we consider different choices of bandwidth. Supplementary Figure 8 presents histograms of estimates of β_1 for low-bias and high-bias schools for bandwidths ranging from 20 to 300 days in 10 day increments. The estimates are all clustered around the baseline estimates of 0.39 for low-bias schools and 0.33 for high-bias schools, showing that our conclusions are robust to the choice of the bandwidth.

C Supplementary Discussion

C.1 Level of Aggregation and Exposure vs. Friending Bias

As we discuss in the main text, the distinction between friending bias and exposure depends on the level at which one measures exposure. For example, when measuring exposure and bias at the school level, tracking to different classes may result in higher observed friending bias at the school level; however, that friending bias might be the result of lower exposure (lack of integration by SES) at the classroom level. Similar phenomena arise in other settings: for instance, ZIP codes that exhibit high friending bias may be highly segregated by income across blocks within the ZIP code. Indeed, we find that counties with higher levels of residential income segregation have higher levels of mean friending bias, even after controlling for the exposure of low-SES individuals to high-SES individuals in their groups, suggesting that friending bias is partly the result of residential income segregation within ZIP codes.

Despite these observations, the efficacy of interventions in increasing economic connectedness depends on the importance of exposure and friending bias at the units of aggregation we analyze, since those are often units of interest for policy makers (e.g., high schools, colleges, and workplaces). For example, if low-SES students have few high-SES friends in their high schools because of a lack of exposure at the high school level, then integration across schools could be effective in increasing EC. But if low-SES students have few high-SES friends even in schools that are socioeconomically diverse, other within-school interventions (such as changes in tracking) may be necessary to increase EC.

C.2 Relationship Between Exposure and Average Incomes Across Areas

In the main text, we show that mean levels of high-SES exposure and the overall share of high-SES people in a given ZIP code or county are highly correlated with each other. Although these correlations are intuitive, they are not mechanical because exposure for low-SES individuals is constructed at the group level, based on, e.g., the specific high schools or recreational groups in which low-SES people participate.

Differences between exposure and the local share of high-SES (or high-income) people provide new information about the degree to which groups in an area are integrated by SES. In particular, two ZIP codes that have similar shares of high-SES people overall but different levels of high-SES exposure must differ in their degree of income segregation across groups; that is, the extent to which low- and high-SES residents attend the same schools, work at the same employers, participate in the same recreational groups, etc. Relatedly, note that high-SES and low-SES individuals who live in the same ZIP code may have different levels of high-SES exposure because they may be members of different groups within the same area.

C.3 Relationship Between Upward Mobility and Friending Bias

Our finding that upward mobility is strongly related to friending bias echoes the findings in Figure 6 and Table 4a of Chetty et al. (2022), where we showed that economic connectedness strongly predicts economic mobility even conditional on income levels in the areas where low-SES people live. Here, we further establish that controlling for SES in the specific *groups* that low-SES individuals belong to (i.e., their high schools, religious groups, etc.)—which differs from overall SES in the areas they live in for the reasons discussed in the preceding subsection—does not affect the relationship between economic connectedness and upward mobility.

C.4 Friending Bias at Evanston Township High School: Ethnographic Evidence

The high degree of friending bias at Evanston Township High School (ETHS) observed in our data mirrors historical ethnographic accounts of racial and socioeconomic segregation at the school (Barr 2014). It is also consistent with more recent discussions within the student body. For instance, the student newspaper *The Evanstonian* has frequently reported on racial and socioeconomic disconnections within the student body. In one newspaper article, Jacobs and Martinez-Olsen (2019) highlight socioeconomic disparities in access to extra-curricular activities, arguing that the high cost of prom tickets prevents students from low-income families from attending: "(Prom) is a once in a lifetime opportunity so you'll go all out. It's said to be "the time of your life" but not all students have access to that kind of money."

Another article in *The Evanstonian* describes the efforts of a student group called the Paw Patrol—which seeks to foster school spirit by organizing student cheering at high school sporting events—to increase across-group interaction at ETHS (Dain et al. 2021): "In terms of diversity, Paw Patrol has been successful in getting a range of students involved. [...] However, diversity and integration are two commonly confused terms with entirely separate meanings. Diversity applies solely to demographics, whereas integration investigates the interpersonal connections across those demographics. Paw Patrol has undergone improvement regarding diversity but still requires growth in terms of integration. 'I definitely feel like there is segregation, and we've talked about it,' [student leader Maya] Wallace explains."

Supplementary References

Barr, Mary (2014). *Friends Disappear: The Battle for Racial Equality in Evanston.* University of Chicago Press.

Bush, Melodye and Kyle Zinth (2011). *Kindergarten Entrance Ages: A 35 Year Trend Analysis.*

Chetty, Raj, John N. Friedman, Nathaniel Hendren, Maggie R Jones, and Sonya R Porter (2018). *The Opportunity Atlas: Mapping the Childhood Roots of Social Mobility.* Working Paper 25147. National Bureau of Economic Research.

Chetty, Raj, John N. Friedman, Emmanuel Saez, Nicholas Turner, and Danny Yagan (2020). "Income Segregation and Intergenerational Mobility Across Colleges in the United States". *Quarterly Journal of Economics* 135.3, pp. 1567–1633.

Chetty, Raj et al. (2022). "Social Capital I: Measurement and Associations with Economic Mobility". *Nature.*

Dain, Lauren, Jessica Sehgal, Meg Houseworth, Bridget Baker, Stella Israelite, Sophie Milner-Gorvine, and Zoe Kaufman (2021). "ETHS activities and spaces grapple with inequities". *The Evanstonian.*

Elder, Todd E. and Darren H. Lubotsky (2009). "Kindergarten Entrance Age and Children's Achievement: Impacts of State Policies, Family Background, and Peers". *Journal of Human Resources* 44.3, pp. 641–683.

Jacobs, Caroline and Aldric Martinez-Olsen (2019). "Racial and socioeconomic implications in how ETHS lost its spirit". *The Evanstonian.*

Pew Research Center (2014). *Religious Landscape Study.* Tech. rep.

U.S. Census Bureau (2017). *2012–2016 American Community Survey 5-Year Estimates.* Tech. rep.

SUPPLEMENTARY TABLE 1: EC, Exposure, and Bias Across High Schools and Colleges Using Parental vs. Own SES Ranks

	Economic Connectedness		Exposure		Friending Bias	
Variables	High School (1)	College (2)	High School (3)	College (4)	High School (5)	College (6)
Corr. Using Par vs Own Rank	0.83	0.84	0.84	0.86	0.59	0.61
Mean Using Parent SES Rank	0.92	1.11	0.93	1.13	0.01	0.02
SD Using Parent SES Rank	0.31	0.28	0.31	0.28	0.04	0.03
Mean Using Own SES Rank	0.92	1.22	0.96	1.27	0.03	0.04
SD Using Own SES Rank	0.33	0.36	0.34	0.35	0.06	0.06

Notes: This table compares estimates of economic connectedness, high-SES exposure, and friending bias for high schools and colleges computed using individuals' own SES ranks in adulthood vs. parental SES ranks. We calculate EC, exposure, and friending bias within schools and colleges as described by equations 14, 15 and 16 in Supplementary Information B.5. We restrict the sample to the schools and colleges shown in Figure 5. The first row shows the weighted correlation of the relevant statistics across schools/colleges when computed using parental vs. own SES, weighting by the number of below-median-parental-SES students in each high school/college born between 1990 and 2000. The second and third rows show the mean and standard deviation of the relevant statistic across schools/colleges computed using parental SES rank; the fourth and fifth rows replicate these statistics using own SES rank. When using parental SES rank, we average over below-median-SES individuals born between 1990 and 2000 whom we can link to parents because parental linkage rates are highest for those cohorts. When using own SES rank, we use all below-median-SES individuals born between 1986 and 1996.

SUPPLEMENTARY TABLE 2: School Entrance Cutoffs by State and Birth Cohort

State	Birth Cohorts	Cutoff Date	State	Birth Cohorts	Cutoff Date
AL	1976–1985	1-Oct	MO	1976–1979	1-Oct
	1986–1998	1-Sep		1982–1991	1-Jul
AK		N/A		1992–1998	1-Aug
AZ	1987–1998	1-Sep	MT	1985–1998	10-Sep
AR	1976–1992	1-Oct	NE	1976–1998	15-Oct
	1993–1998	15-Sep	NV	1979–1998	30-Sep
CA	1976–1998	2-Dec	NH	1988–1995	1-Oct
CO	1976–1998	15-Sep	NJ	1979–1998	1-Oct
CT	1976–1998	30-Sep	NM	1976–1998	1-Sep
DC		N/A	NY	1976–1998	1-Dec
DE	1980–1987	31-Dec	NC	1976–1998	15-Oct
	1991–1998	31-Aug	ND	1976–1989	31-Aug
FL	1979–1998	1-Sep	OH	1976–1998	30-Sep
GA	1976–1998	1-Sep	OK	1976–1998	1-Sep
HI	1976–1998	31-Dec	OR	1976–1979	15-Nov
ID	1976–1984	1-Oct		1981–1998	1-Sep
	1988–1998	1-Sep	PA	1983–1998	1-Sep
IL	1976–1980	1-Dec	RI	1976–1998	31-Dec
	1983–1998	1-Sep	SC	1976–1989	1-Nov
IN	1980–1984	1-Sep		1990–1998	1-Sep
	1987–1992	1-Jun	SD	1976–1980	1-Sep
IA	1976–1998	15-Sep	TN	1976–1978	31-Oct
KS	1976–1998	1-Sep		1980–1998	30-Sep
KY	1976–1998	1-Oct	TX	1976–1998	1-Sep
LA	1976–1980	31-Dec	UT	1976–1977	1-Nov
	1984–1998	30-Sep		1978–1998	2-Sep
ME	1976–1998	15-Oct	VT	1976–1985	31-Dec
MD	1976–1997	31-Dec	VA	1976–1980	31-Dec
MA	1980–1998	1-Sep		1988–1998	30-Sep
MI	1976–1998	1-Dec	WA	1982–1998	31-Aug
MN	1976–1998	1-Sep	WV	1976–1978	1-Nov
MS	1976–1998	1-Sep		1980–1998	31-Aug
			WI	1976–1998	1-Sep
			WY	1976–1980	15-Sep

Notes: This table reports the school entrance cutoff dates by state and year that we use for the quasi-experimental cross-cohort and regression discontinuity analyses in Figures 6 and Extended Data Figure 4. We obtain these cutoffs from Elder and Lubotsky (2009) and Bush and Zinth (2011). The cutoffs determine whether children are assigned to earlier or later school cohorts based on their dates of birth.

SUPPLEMENTARY TABLE 3: Summary Statistics on Group and Friendship Assignment

A. Summary Statistics on Assignment to Groups

	Share Users Assigned			Share with > 1 Group (conditional on assignment)	Mean Users per Group	S.D. of Users per Group	Number of Groups
	All	Low-SES	High-SES				
	(1)	(2)	(3)	(4)	(5)	(6)	(7)
Religious Group	17.9%	16.0%	19.8%	38.4%	39	64	332,042
College	42.9%	24.5%	61.3%	17.8%	530	821	2,921
Employer	20.9%	17.0%	24.7%	9.5%	45	881	334,388
High School	74.9%	67.0%	82.8%	3.3%	115	134	24,322
Neighborhood	100.0%	100.0%	100.0%	0.0%	2,484	3,750	29,062
Rec. Group	29.8%	22.7%	37.0%	45.9%	17	48	1,291,559

B. Overlap of Friendship Assignments Across Settings

	Rel. Group	College	Employer	High School	Neighborhood	Rec. Group	Uniquely Assigned
Religious Group	100.0%	1.8%	0.3%	12.0%	33.2%	3.8%	48.8%
College	0.3%	100.0%	0.4%	27.6%	7.7%	0.7%	63.3%
Employer	0.4%	2.4%	100.0%	5.0%	14.2%	1.0%	77.1%
High School	0.3%	3.8%	0.1%	100.0%	12.8%	0.7%	82.3%
Neighborhood	1.7%	2.1%	0.6%	25.4%	100.0%	2.0%	68.2%
Recreational Group	3.5%	3.2%	0.7%	25.6%	36.1%	100.0%	30.9%

Notes: Panel A shows summary statistics regarding the assignment of individuals to each of the six settings (shown in the rows) that we analyze. The sample used in this table includes all individuals between the ages of 25 and 44 as of May 28, 2022 who reside in the United States, have been active on the Facebook platform at least once in the previous 30 days, have at least 100 U.S.-based Facebook friends, have a non-missing residential ZIP code, and for whom we are able to allocate at least one friend to a setting using the algorithm described in the Variable Definitions section of Methods. Column 1 lists the share of individuals in our primary analysis sample assigned to one or more groups in the relevant setting (e.g., the fraction of individuals assigned to a religious group in row 1). Columns 2 and 3 replicate column 1 for low-SES and high-SES individuals, respectively. Column 4 shows the share of users assigned to more than one group given that they are assigned to at least one group in a setting. As an example, for religious groups, this is the share of users assigned to more than one religious group conditional on being assigned at least one religious group. Columns 5 and 6 show means and standard deviations of group size (Facebook users in the analysis sample) by setting; for high schools and colleges, we report the number of users per cohort. Column 7 shows the number of unique groups within each setting. Panel B reports statistics that characterize the share of friendships allocated to multiple settings. The off-diagonal elements show the fraction of friendships made in the setting shown in a given row that are also assigned to the setting shown in the column; for instance, 1.8% of the friendships assigned to religious groups are also assigned to colleges. The diagonal elements correspond to own-group pairs and are thus 100%. The last column shows a lower bound on the fraction of friendships that can be unambiguously assigned to a given group type. This bound is calculated by subtracting the off-diagonal elements in each row from 100%; intuitively, this bound assumes that all intersections of three or more groups are empty.

SUPPLEMENTARY FIGURE 1: Predictors of Friending Bias across Settings

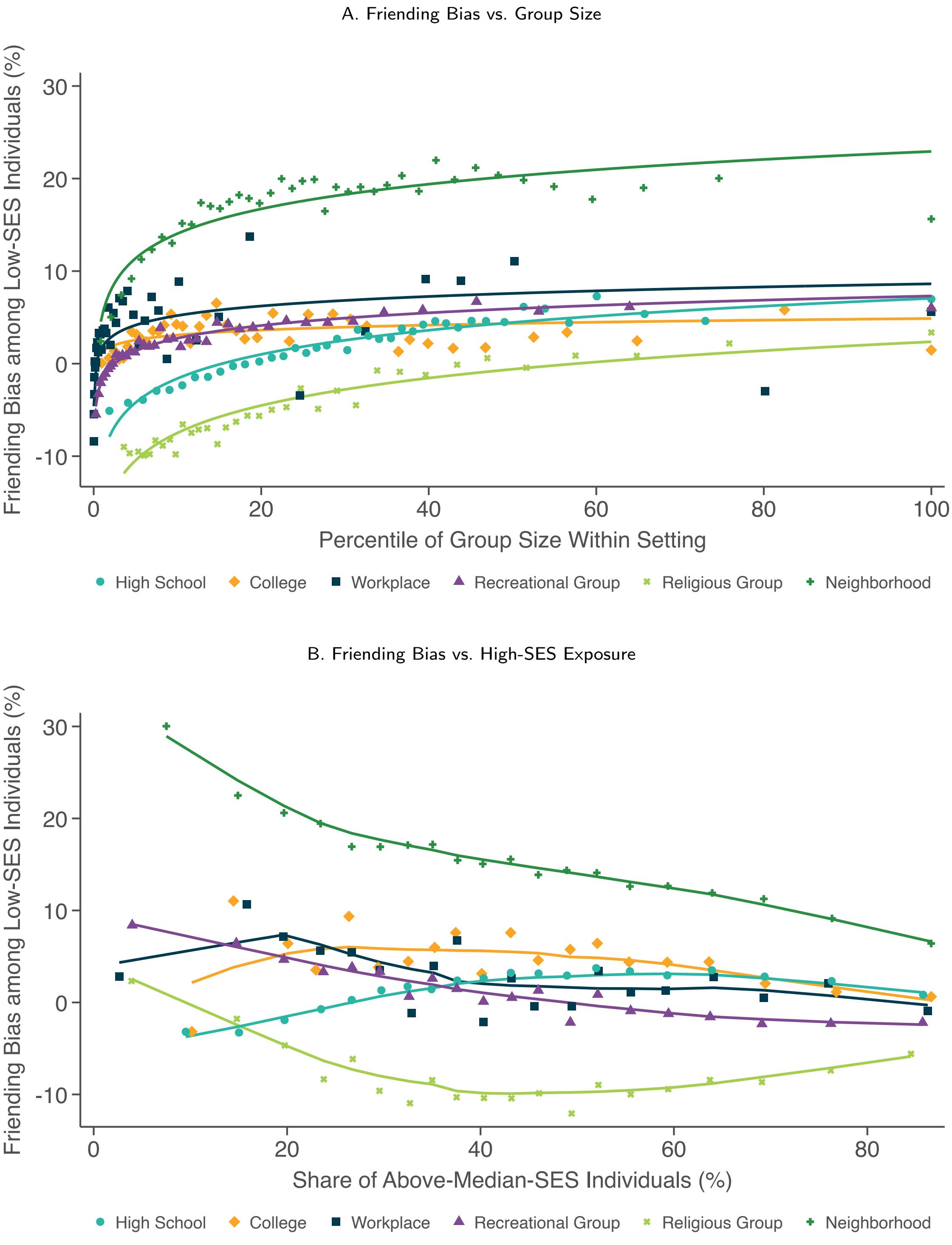

Notes: Panel A plots mean friending bias among below-median-SES individuals vs. group size, measured as a percentile in the within-setting group size distribution, weighting by the number of low-SES individuals. Panel B presents a binned scatter plot of friending bias vs. the share of above-median SES individuals in the group, again weighting by the number of low-SES individuals. Both friending bias and the share of high-SES individuals are computed using the 1986-1996 birth cohorts and using individuals' own SES. Unlike Extended Data Figures 1 and 2 and Supplementary Figure 3, we do not impose any restrictions on the set of high schools and colleges included in this figure.

SUPPLEMENTARY FIGURE 2: Friending Bias and Exposure by High School and College, Based on Own SES

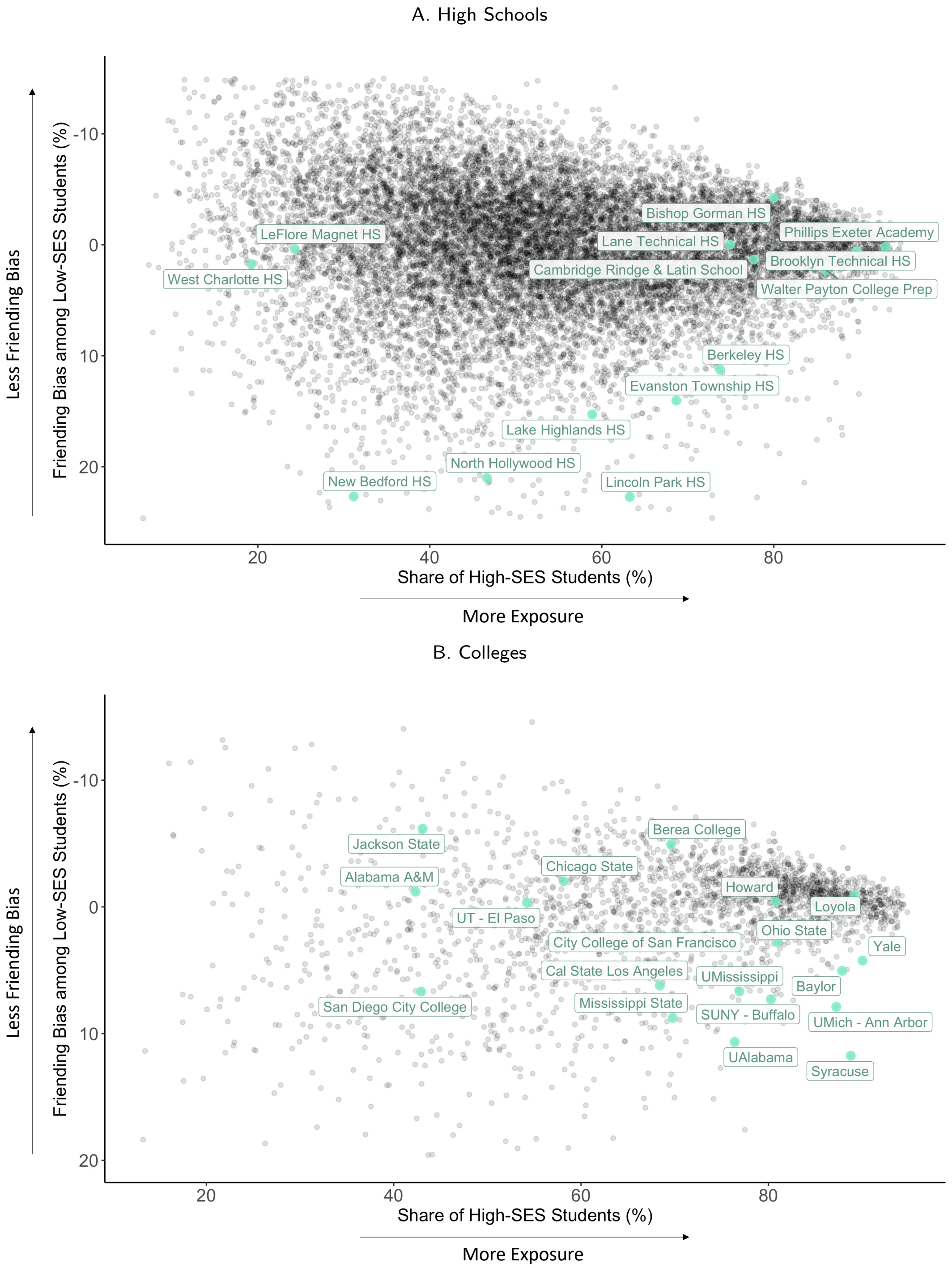

Notes: This figure replicates Figure 5 using own (post-high-school) SES rank instead of parental SES rank. In this figure, we focus on the 1986-1996 birth cohorts (aged between 26–36 in 2022) to obtain more reliable measures of SES ranks in adulthood. We label the same schools that are labeled in Figure 5, unless they fail to meet the minimum threshold of 100 low-SES and 100 high-SES Facebook users when using individuals' own SES ranks. For example, some elite private high schools meet this threshold for parental SES but not own SES, as their students' own SES in adulthood tends to be significantly higher than that of their parents.

SUPPLEMENTARY FIGURE 3: Predictors of Friending Bias in Colleges

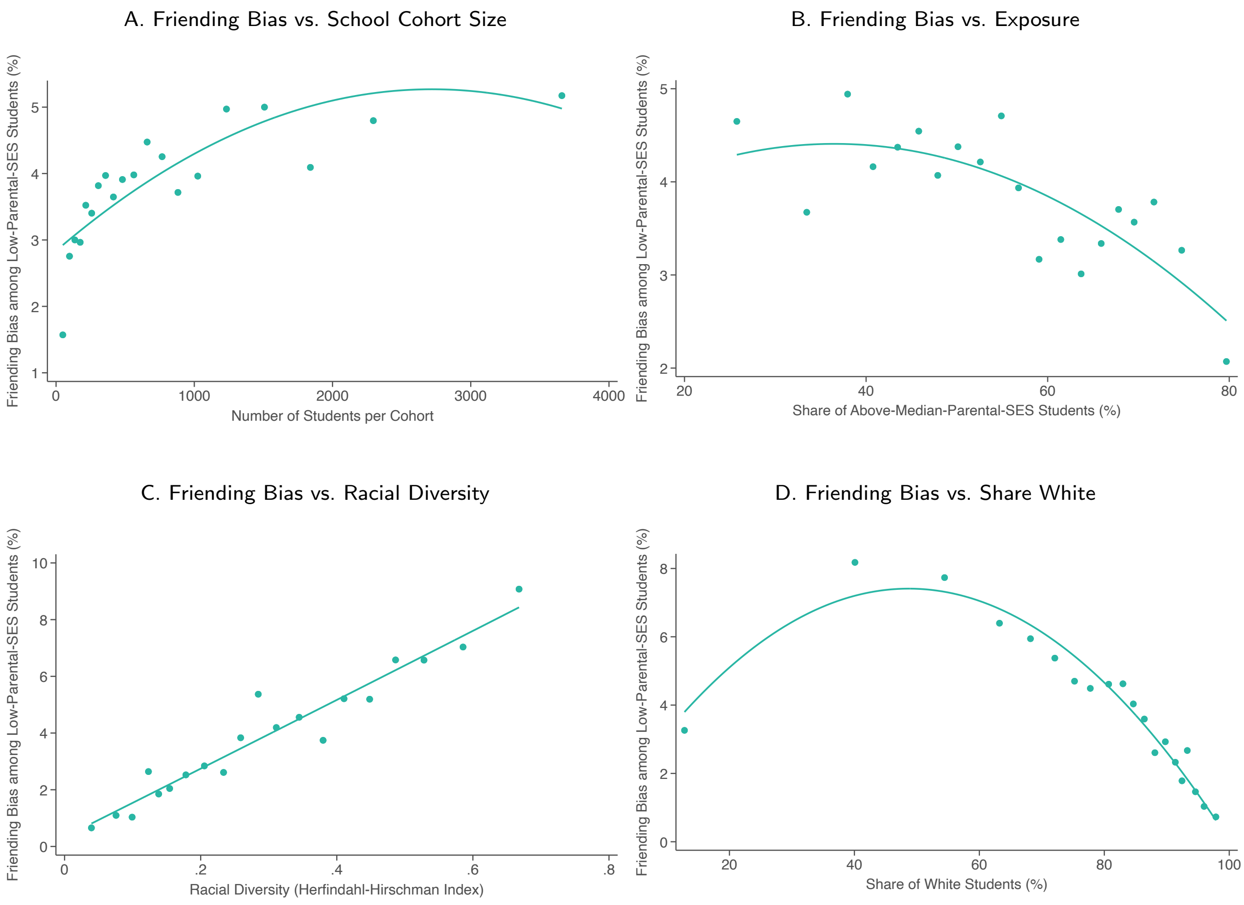

Notes: This figure replicates Extended Data Figures 1c-f in colleges instead of high schools. See notes to Extended Data Figure 1 for details.

SUPPLEMENTARY FIGURE 4: Friending Rates and Determinants of Economic Connectedness, Correcting for Underreporting of Group Memberships

A. Friending Rates

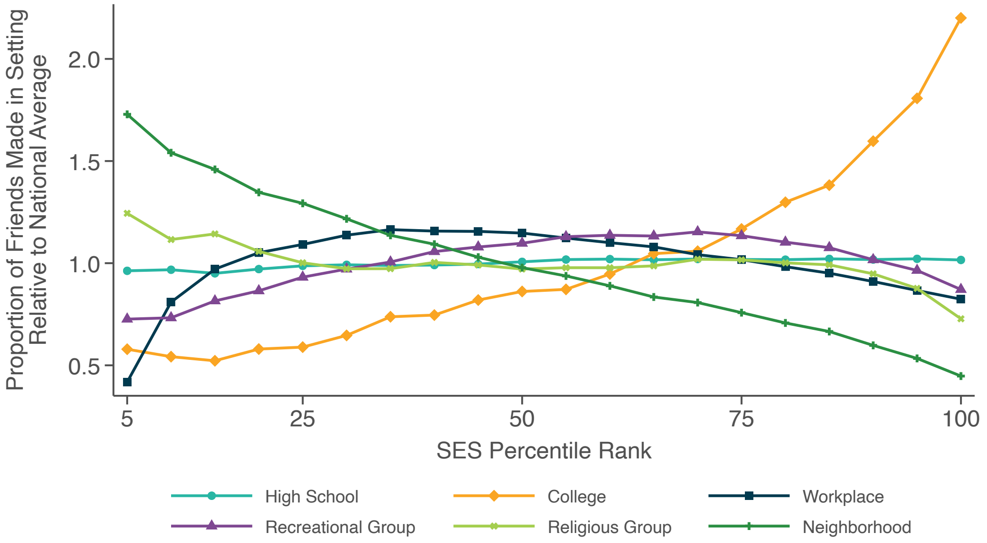

B. Decomposing Economic Connectedness

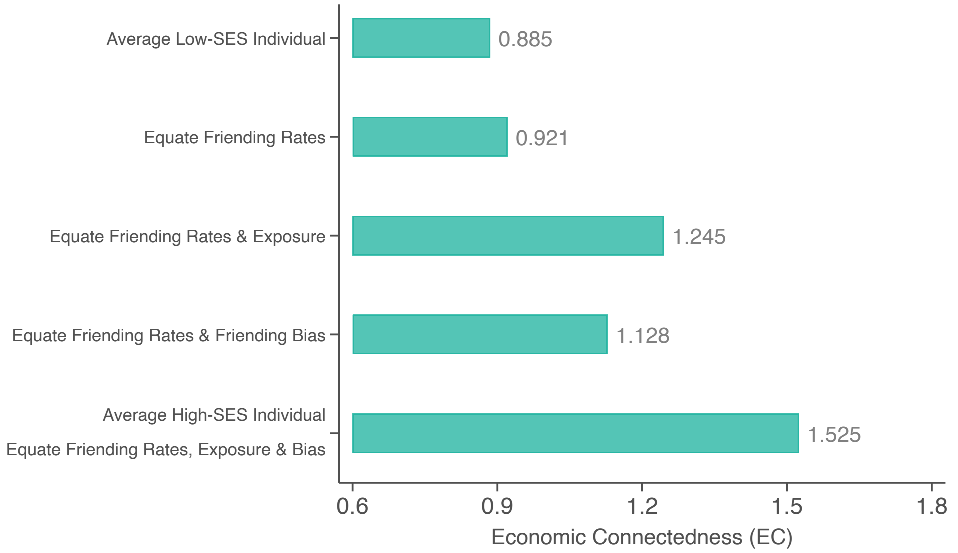

Notes: Panel A replicates Figure 1 and Panel B replicates Figure 3a correcting for underreporting of group memberships by Facebook users using the algorithm described in Supplementary Information B.3, which inflates the raw share of friends made across groups by group membership rates estimated from external data.

SUPPLEMENTARY FIGURE 5: Autocorrelation of EC by Birth Cohort Across High Schools and Colleges

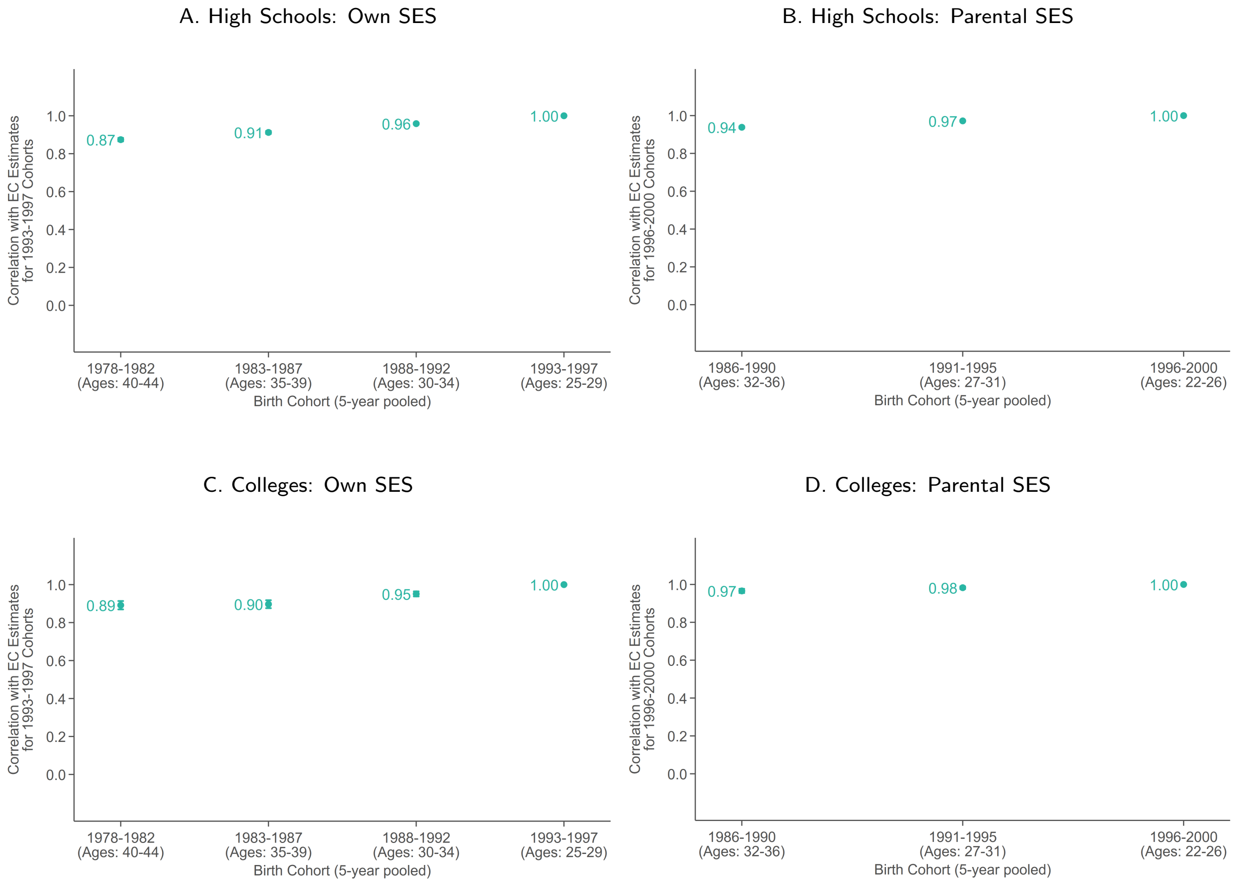

Notes: Panel A plots the serial correlation across high schools of EC estimated using individuals' own SES different birth cohorts. Each point shows the correlation between estimates of EC based on a given 5 year birth cohort range, starting from 1978-1982, with the reference cohort group of 1993-1997. We report the ages when SES is measured (in 2022) below each cohort group. Panel B replicates Panel A using parental SES, with 5-year intervals starting from the 1986-1990 cohorts and a reference cohort group of 1996-2000. Panels C and D replicate Panels A and B at the college level. All correlations are weighted by the average number of low-SES students in the relevant pair of five-year cohort groups. When analyzing own SES (Panels A and C), we restrict the sample to schools that meet the following size restrictions over the relevant 5-year intervals: more than 75 low-SES students, more than 75 high-SES students, and more than 200 total students. When analyzing parental SES (Panels B and D), we require more than 10 low-SES students, more than 10 high-SES students, and more than 25 total students.

SUPPLEMENTARY FIGURE 6: Causal Effects of Changes in Socioeconomic Integration on Economic Connectedness in High Schools: Sensitivity Analysis

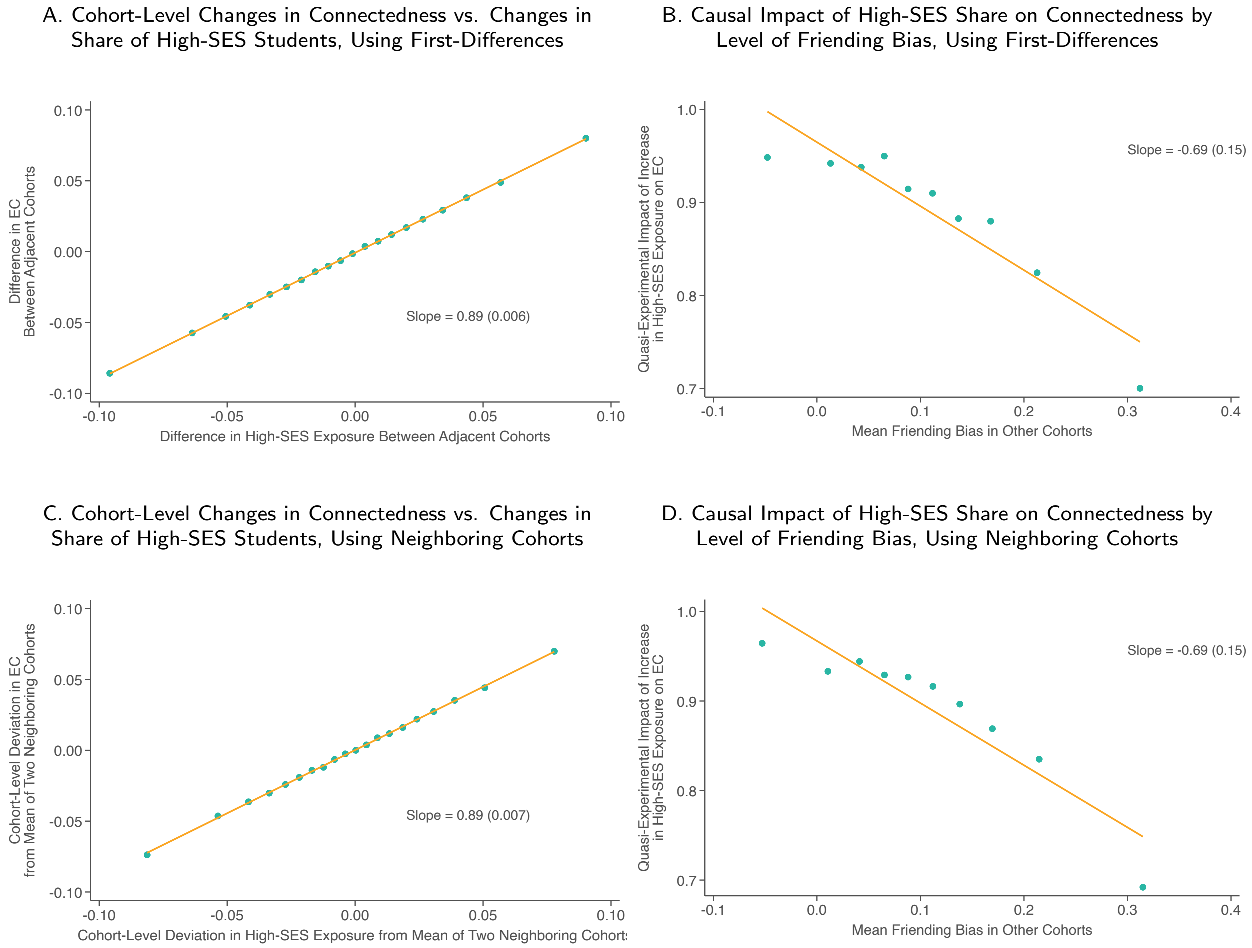

Notes: This figure replicates Figure 6 using alternative measures of changes in exposure and economic connectedness across cohorts. Panels A and B use differences in exposure and economic connectedness between adjacent cohorts, measuring friending bias in cohorts excluding the two adjacent cohorts used to compute changes in exposure and EC. Panels C and D demean exposure and EC using the mean of the two neighboring cohorts, instead of the mean over all other cohorts in the school as in Figure 6. See notes to Figure 6 for further details.

SUPPLEMENTARY FIGURE 7: Balance Tests for Regression Discontinuity Design

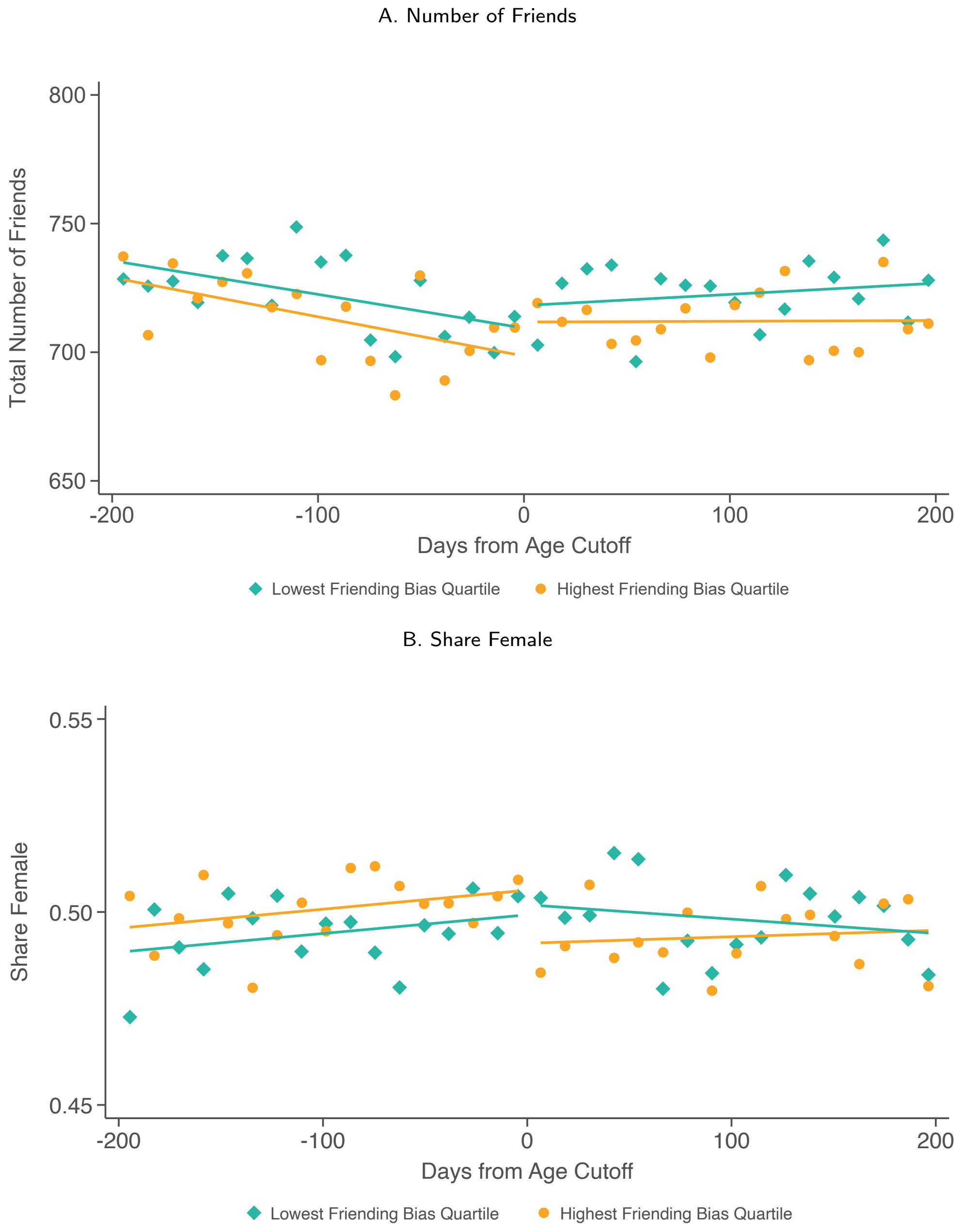

Notes: This figure evaluates the identification assumption underlying the regression discontinuity design by replicating Extended Data Figure 4a using other variables: the total number of friends individuals have in the primary analysis sample (Panel A) and an indicator for being female (Panel B). See notes to Extended Data Figure 4 for details on the construction of this figure.

SUPPLEMENTARY FIGURE 8: RD Estimates of Causal Effects of Exposure on EC, by Bandwidth

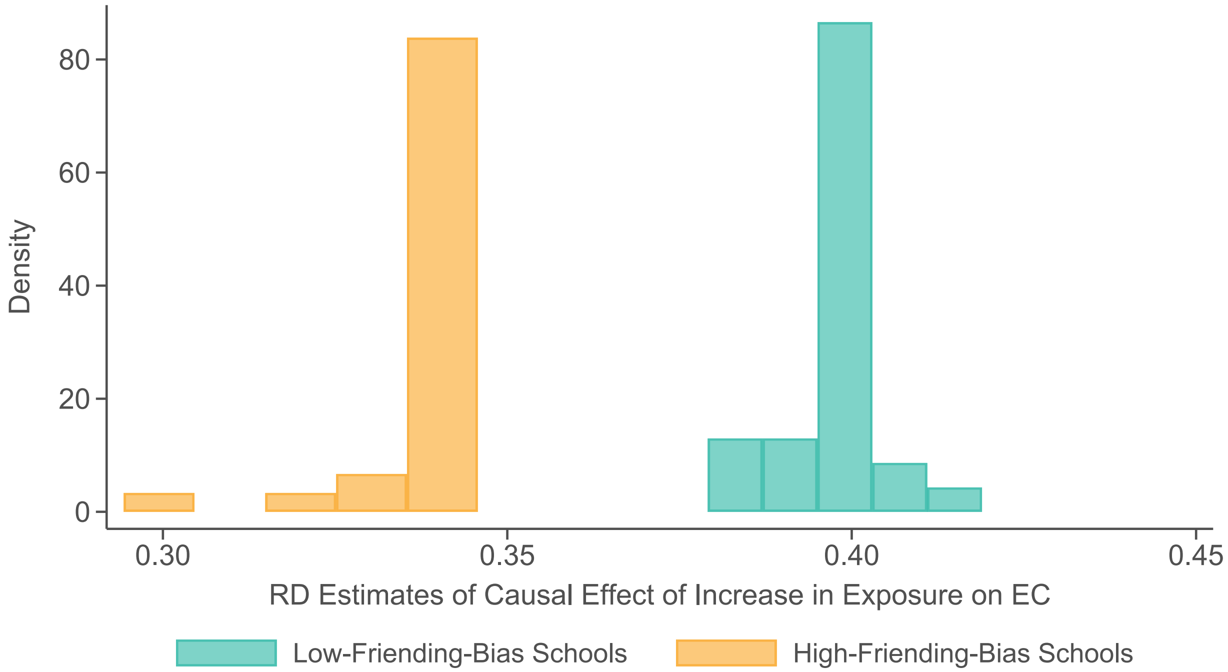

Notes: Extended Data Figure 4a reports regression discontinuity estimates of the causal effect of changes in exposure on EC separately for low-bias and high-bias schools using linear regressions with a bandwidth of 200 days around the school entry cutoff (see Supplementary Information B.6 for the regression specification). This figure presents histograms of estimates from the same regression specification, varying the bandwidth around the school entry cutoff from 20 to 300 days in 10 day increments, separately for low-bias and high-bias schools.
