# Supplementary material for: Social capital II: determinants of economic connectedness
Source: Nature. 2022 Aug 1;608(7921):122–34. doi: 10.1038/s41586-022-04997-3 (PMC9352593; doi:10.1038/s41586-022-04997-3)
Supplement: Supplementary file 2 — Reporting Summary [file 41586_2022_4997_MOESM2_ESM.pdf]

nature portfolio

Corresponding author(s): Raj Chetty
Last updated by author(s): 2022/6/12

Reporting Summary

Nature Portfolio wishes to improve the reproducibility of the work that we publish. This form provides structure for consistency and transparency in reporting. For further information on Nature Portfolio policies, see our Editorial Policies and the Editorial Policy Checklist.

Statistics

For all statistical analyses, confirm that the following items are present in the figure legend, table legend, main text, or Methods section.

n/a	Confirmed	
[ ]	[x]	The exact sample size (*n*) for each experimental group/condition, given as a discrete number and unit of measurement
[x]	[ ]	A statement on whether measurements were taken from distinct samples or whether the same sample was measured repeatedly
[ ]	[x]	The statistical test(s) used AND whether they are one- or two-sided<br>*Only common tests should be described solely by name; describe more complex techniques in the Methods section.*
[ ]	[x]	A description of all covariates tested
[ ]	[x]	A description of any assumptions or corrections, such as tests of normality and adjustment for multiple comparisons
[ ]	[x]	A full description of the statistical parameters including central tendency (e.g. means) or other basic estimates (e.g. regression coefficient) AND variation (e.g. standard deviation) or associated estimates of uncertainty (e.g. confidence intervals)
[x]	[ ]	For null hypothesis testing, the test statistic (e.g. *F*, *t*, *r*) with confidence intervals, effect sizes, degrees of freedom and *P* value noted<br>*Give P values as exact values whenever suitable.*
[x]	[ ]	For Bayesian analysis, information on the choice of priors and Markov chain Monte Carlo settings
[x]	[ ]	For hierarchical and complex designs, identification of the appropriate level for tests and full reporting of outcomes
[x]	[ ]	Estimates of effect sizes (e.g. Cohen's *d*, Pearson's *r*), indicating how they were calculated

Our web collection on statistics for biologists contains articles on many of the points above.

Software and code

Policy information about availability of computer code

Data collection	Researchers were not involved in data collection. The study works with privacy-protected data from Facebook. This project focuses on drawing high-level insights about communities and groups of people, rather than individuals.
Data analysis	We used a server-side analysis script that was designed to automatically process the raw data, strip the data of personal identifiers, and generate aggregated results, which we analyzed to produce the conclusions in this paper. All inferences made as part of this research were created and used solely for the purpose of this research and were not used by Meta (which owns the Facebook platform) for any other purpose. The script then promptly deleted the raw data generated for this project. Software used to produce figures and tables: Stata 17; R 4.1.0

For manuscripts utilizing custom algorithms or software that are central to the research but not yet described in published literature, software must be made available to editors and reviewers. We strongly encourage code deposition in a community repository (e.g. GitHub). See the Nature Portfolio guidelines for submitting code & software for further information.

Data

Policy information about availability of data

All manuscripts must include a data availability statement. This statement should provide the following information, where applicable:
- Accession codes, unique identifiers, or web links for publicly available datasets
- A description of any restrictions on data availability
- For clinical datasets or third party data, please ensure that the statement adheres to our policy

A publicly available dataset, which only includes aggregate statistics on social capital, is available at www.socialcapital.org. We use methods from the differential

privacy literature to add noise to these aggregate statistics to protect privacy while maintaining a high level of statistical reliability; see www.socialcapital.org for further details on these procedures.

Field-specific reporting

Please select the one below that is the best fit for your research. If you are not sure, read the appropriate sections before making your selection.

☐ Life sciences ☒ Behavioural & social sciences ☐ Ecological, evolutionary & environmental sciences

For a reference copy of the document with all sections, see nature.com/documents/nr-reporting-summary-flat.pdf

Behavioural & social sciences study design

All studies must disclose on these points even when the disclosure is negative.

Study description	We use privacy-protected data from Facebook to analyze the determinants of cross-class interaction, distinguishing between two mechanisms: exposure (the extent to which people with low versus high socioeconomic status (SES) participate in the same groups) and friending bias conditional on exposure (the tendency for low-SES people to befriend high-SES people at lower rates even conditional on exposure).
Research sample	Our primary analysis sample was constructed by limiting the Facebook data to users aged between 25 and 44 years who reside in the United States, were active on the Facebook platform at least once in the previous 30 days, had at least 100 US-based Facebook friends, and had a non-missing residential ZIP code as of 28 May, 2022. We further restrict attention to the subset of individuals for whom we can allocate at least one friendship to the group in which it was formed. Our final analysis sample consisted of 70.3 million Facebook users, who constitute 82% of the US population between ages 25 and 44 years (based on a comparison to the 2014–2018 American Community Survey (ACS)). We focused on the 25–44-year age range because previous work has documented that its Facebook usage rate is above 80%, higher than for other age groups.
Sampling strategy	All individuals in the Facebook data who met the sample criteria were included in the analysis.
Data collection	Researchers were not involved in data collection. The study works with privacy-protected data from Facebook.
Timing	We use a snapshot of Facebook data as of 28 May, 2022.
Data exclusions	We did not exclude any individuals who met the Research Sample criteria.
Non-participation	No participants dropped out or declined participation.
Randomization	The data is observational and there is no random variation. We discuss how our correlational results should be interpreted.

Reporting for specific materials, systems and methods

We require information from authors about some types of materials, experimental systems and methods used in many studies. Here, indicate whether each material, system or method listed is relevant to your study. If you are not sure if a list item applies to your research, read the appropriate section before selecting a response.

Materials & experimental systems

n/a	Involved in the study	
☒	☐	Antibodies
☒	☐	Eukaryotic cell lines
☒	☐	Palaeontology and archaeology
☒	☐	Animals and other organisms
☐	☒	Human research participants
☒	☐	Clinical data
☒	☐	Dual use research of concern

Methods

n/a	Involved in the study	
☒	☐	ChIP-seq
☒	☐	Flow cytometry
☒	☐	MRI-based neuroimaging

Human research participants

Policy information about studies involving human research participants

Population characteristics	See above
Recruitment	Facebook users meeting the sample criteria were included in the study.
Ethics oversight	The project was approved under Harvard University IRB 17-1692.

Note that full information on the approval of the study protocol must also be provided in the manuscript.
